# MicroRNA Expression Profile in Bovine Granulosa Cells of Preovulatory Dominant and Subordinate Follicles during the Late Follicular Phase of the Estrous Cycle

**DOI:** 10.1371/journal.pone.0125912

**Published:** 2015-05-19

**Authors:** Samuel Gebremedhn, Dessie Salilew-Wondim, Ijaz Ahmad, Sudeep Sahadevan, Md Munir Hossain, Michael Hoelker, Franca Rings, Christiane Neuhoff, Ernst Tholen, Christian Looft, Karl Schellander, Dawit Tesfaye

**Affiliations:** 1 Institute of Animal Science, Dept. Animal Breeding and Husbandry, University of Bonn, Endenicher Allee 15, 53115 Bonn, Germany; 2 Department of Animal Breeding & Genetics, Bangladesh Agricultural University, Mymensingh 2202, Bangladesh; China Agricultural University, CHINA

## Abstract

In bovine, ovarian follicles grow in a wave-like fashion with commonly 2 or 3 follicular waves emerging per estrous cycle. The dominant follicle of the follicular wave which coincides with the LH-surge becomes ovulatory, leaving the subordinate follicles to undergo atresia. These physiological processes are controlled by timely and spatially expressed genes and gene products, which in turn are regulated by post-transcriptional regulators. MicroRNAs, a class of short non-coding RNA molecules, are one of the important posttranscriptional regulators of genes associated with various cellular processes. Here we investigated the expression pattern of miRNAs in granulosa cells of bovine preovulatory dominant and subordinate follicles during the late follicular phase of bovine estrous cycle using Illumina miRNA deep sequencing. In addition to 11 putative novel miRNAs, a total of 315 and 323 known miRNAs were detected in preovulatory dominant and subordinate follicles, respectively. Moreover, in comparison with the subordinate follicles, a total of 64 miRNAs were found to be differentially expressed in preovulatory dominant follicles, of which 34 miRNAs including the miR-132 and miR-183 clusters were significantly enriched, and 30 miRNAs including the miR-17-92 cluster, bta-miR-409a and bta-miR-378 were significantly down regulated in preovulatory dominant follicles. In-silico pathway analysis revealed that canonical pathways related to oncogenesis, cell adhesion, cell proliferation, apoptosis and metabolism were significantly enriched by the predicted target genes of differentially expressed miRNAs. Furthermore, Luciferase reporter assay analysis showed that one of the differentially regulated miRNAs, the miR-183 cluster miRNAs, were validated to target the 3´-UTR of *FOXO1* gene. Moreover *FOXO1* was highly enriched in granulosa cells of subordinate follicles in comparison with the preovulatory dominant follicles demonstrating reciprocal expression pattern with miR-183 cluster miRNAs. In conclusion, the presence of distinct sets of miRNAs in granulosa cells of preovulatory dominant and subordinate follicles supports the potential role of miRNAs in post-transcriptional regulation of genes involved in bovine follicular development during the late follicular phase of the estrous cycle.

## Introduction

Bovine follicular development is a highly orchestrated, periodic and gonadotropin-dependent process which starts with the activation of resting follicles gradually leading to the growth and development of a preovulatory follicle accompanied by sequential differentiation of oocyte and the surrounding somatic cells [1]. In cattle, recruitment of growing follicle, selection and growth of leading follicles, ovulation of the preovulatory dominant follicle and degeneration of anovulatory subordinate follicles undertake in a wave-like fashion with typically 2 or 3 follicular waves per estrous cycle [2,3]. The first follicular wave emerges soon after ovulation, whereas the second and third follicular waves start to emerge 8–9 and 15–16 days post ovulation, respectively [4]. The dominant follicle of the wave which coincides with a sharp rise of luteinizing hormone (LH-surge) becomes ovulatory, while the remaining follicles of both the first and second waves eventually undergo follicular atresia [4].

Follicular recruitment, selection, dominance and ovulation are tightly regulated by endocrine and paracrine hormones, among which the follicle stimulating hormone (FSH) and luteinizing hormone (LH) play crucial roles [5]. Throughout the estrous cycle granulosa cells undergo several developmental changes. During the early stage of the cycle, granulosa cells of small growing follicles secret inhibin and acquire follicle stimulating hormone receptors (*FSHR*). Granulosa cells of dominant follicles acquire LH receptors (*LHCGR*) and secrete more estradiol than the subordinate follicles, triggering the LH surge that allows it to develop into preovulatory follicle. These synergistically coordinated actions of hormones induce tremendous morphological reorganization and functional changes in the oocyte and its companion somatic cells, which lead to cellular differentiation and give rise to a developmentally competent oocyte.

Cellular differentiation that occurs during follicular development are regulated by the expression and interaction of multitude of genes in spatio-temporal manner in different compartment of the follicle: granulosa cells [6,7], theca cells [8], follicular fluid and oocyte [9]. Nevertheless, understanding the post-transcriptional regulation of genes in follicular cells at different stages of follicular development would help us to understand the molecular mechanisms underlying follicular recruitment, selection, ovulation and follicular atresia in more depth.

MicroRNAs (miRNAs) are class of endogenous short non-coding RNA molecules, ~22-nucleotides (nt) long that post-transcriptionally regulate expression of genes by either degrading the mRNA or translational repression [10]. Previously, we have shown the presence of distinct set of miRNAs in bovine ovary [11] and spatio-temporal expression of miRNAs in bovine oocytes, cumulus cells and preimplantation embryos [12,13]. Interestingly, both bovine oocyte and cumulus cells were found to be interdependent on each other for signals affecting the activity of miRNAs [12].

Similarly, involvement of miRNAs in mouse LH-induced luteinization and ovulation was demonstrated in mural granulosa cells after LH/hCG stimulation [14]. In addition, association of selected candidate miRNAs with follicular selection, maturation and ovulation in mare granulosa cells and follicular fluid derived from ovulatory dominant, largest subordinate and anovulatory dominant follicles has been demonstrated by Schauer *et al*. [15]. Despite the fact that follicular development in mammalian species is regulated by spatio-temporal expression of genes [9], little is known about the abundance of larger set of miRNAs in follicular cells.

Recently, we reported the expression pattern of miRNAs in granulosa cells of subordinate and dominant follicles and the possible association with follicular recruitment, selection and dominance during the early luteal phase of bovine estrous cycle [16]. Nonetheless, information on the abundance of regulatory miRNAs in granulosa cells of bovine preovulatory dominant and subordinate follicles during the follicular phase of the estrous cycle is missing. Thus, we aimed to examine differences in miRNA abundance in bovine granulosa cells derived from preovulatory dominant and subordinate follicles obtained at day 19 of the bovine estrous cycle. Moreover, the potential role of miRNAs in ovulation and follicular atresia is elucidated by predicting significantly enriched canonical pathways by predicted target genes of differentially expressed miRNAs.

## Materials and Methods

### Experimental animals and estrous synchronization

Handling of experimental animals was in accordance with the 1972 German law of rules and regulations of animal protection. The experiment was licensed by the animal welfare office of the University of Bonn with proposition number 84–02.05.20.12.075. Healthy Simmental heifers (n = 7) aged between 15 and 20 months and weight range of 380 to 500 kg were used for this study. Animals were housed in the same herd and farm with free-stall barn fitted with slotted floors and cubicles lined with rubber mats. Heifers were synchronized according to standard synchronization protocols as previously described [16,17]. Briefly; all experimental heifers were pre-synchronized by intramuscular (IM) administration of 500 mg of prostaglandin 2-alpha (PGF_2α_)-analogue; cloprostenol (Estrumate, Munich, Germany) twice within 11 days. Two days after each of the PGF_2α_ treatments animals received 0.02 mg of gonadotropin releasing hormone (GnRH)-analogue; buserelin (Receptal; Intervet, Boxmeer, The Netherlands). Common signs of oestrus were visually monitored three times a day. The day at which animals exhibit oestrus was considered as day 0. Finally, synchronized heifers were slaughtered 19 days after the onset of oestrus using a standard scarifying procedure at a local slaughter house.

### Ovary collection and Follicle categorization

Ovaries were retrieved and immediately transported in thermos flask containing warm (37°C) physiological saline solution (0.9% NaCl) solution. Up on arrival, ovaries were repeatedly washed with warm (0.9% NaCl), rinsed in 70% ethanol for 30 seconds and washed again three times with warm saline solution. Ovaries were visually inspected for the presence of one bigger preovulatory dominant follicle and several other subordinate follicles. Ovary samples of 2 cows lacking preovulatory dominant follicles were excluded from the experiment. Follicles were carefully isolated from ovaries by dissecting using scissors and forceps and were categorized according their external surface diameter as previously described in Fortune et al. [3] and Ireland et al. [18] with minor modifications. Briefly; follicles with external surface diameter of 12 mm and above were categorized as preovulatory dominant and follicles with surface diameter ≤ 11 mm were considered as subordinate.

### Granulosa cells, Theca cells, Cumulus-Oocyte-Complex and Follicular Fluid collection

Each of the retrieved preovulatory dominant and subordinate follicles were dissected into two halves and follicular contents were released into a sterile plastic petri dish. Cumulus-oocyte-complexes (COC) were recovered, transferred into separate tubes and stored -80°C for further analysis. Follicular fluid samples devoid of COC were transferred into 15 ml sterilized falcon tubes and centrifuged at 750*xg* for 7 minutes to separate the granulosa cells pellet from follicular fluid supernatant. Follicular fluid samples were transferred into separate tube and stored at -80°C until further use. Granulosa cells pellets were washed twice with PBS (without Ca²^+^ and Mg²^+^) and stored at -80°C for further analysis.

Similarly, theca cell layers were gently scraped from both the preovulatory dominant and the subordinate follicles as previously described [19] with minor modification. Briefly; follicle halves were examined under dissecting microscope and theca cell layers (theca interna and theca externa) were gently peeled away from the basal membrane using forceps. Theca cell samples were repeatedly washed with PBS (without Ca²^+^ and Mg²^+^) to avoid granulosa cells contamination. Finally, theca cell samples were transferred into 0.65 ml sterilized tubes containing RNAlater solution (Sigma-Aldrich, Steinheim, Germany) and stored in -20°C until further processing.

### Total RNA isolation and quality control

Total RNA enriched with miRNAs was isolated from granulosa cells, theca cells and follicular fluid samples of both preovulatory dominant and subordinate follicles using the miRNeasy mini kit (Qiagen GmbH, Hilden, Germany) according to manufacturer’s instruction. On-column DNA digestion was performed to remove genomic DNA contamination using RNase-free DNase (Qiagen GmbH, Hilden, Germany). Similarly, total RNA from COC was isolated using AllPrep DNA/RNA Micro Kit (Qiagen GmbH, Hilden, Germany) following manufacturer´s protocol. RNA concentration and integrity were assessed by NanoDrop 8000 spectrophotometer (NanoDrop technologies, Wilmington, DE, USA) and Agilent 2100 Bioanalyzer (Agilent Technologies, Santa Clara, CA, USA), respectively. Finally, total RNA samples of granulosa cells were pooled to generate biological triplicates and used for library preparation and miRNA deep sequencing. RNA samples from theca cell, COC and follicular fluid were used to determine the expression pattern of selected differentially expressed miRNAs in granulosa cells of preovulatory dominant follicles.

### Purity of isolated Granulosa cells

Purity of isolated granulosa cell was assessed by analysing the presence of granulosa cell specific marker gene (*FSHR*) and absence of theca cell specific marker gene (*CYP17A1)*. For this, specific primers targeting *FSHR*, *CYP17A1* and *GAPDH* were designed using primer3web version 4.0.4 (http://bioinfo.ut.ee/primer3/) (S1 Table). Complementary DNA (cDNA) was synthesized using First Strand cDNA Synthesis Kit (Thermo scientific, MA, and USA) according to manufacturer´s instruction. Briefly; 1 μg of total RNA samples isolated from granulosa cells was mixed with 1 μl of oligo(dT)_18_ were incubated at 65°C for 5 minutes. Nine μl of master mix (4 μl of 5X reaction buffer, 1 μl RiboLock RNase inhibitor, 2 μl of 10 mM dNTP mix and 2 μl of M-MuLV Reverse Transcriptase) was added to the RNA template and incubated at 37°C for 60 minutes. Reactions were terminated by heating at 75°C for 5 minutes. Finally, polymerase chain reaction (PCR) was set with thermocycling conditions of: pre-incubation at 95°C for 5 min, 40 cycles of denaturation at 95°C for 30 s, annealing at 55°C (*FSHR* and *GAPDH*) and 57°C (*CYP17A1*) for 30 s, extension at 72°C for 1 min and final extension at 72°C for 10 min. The PCR product was mixed with loading buffer and loaded into 2% agarose gel stained with Ethidium bromide (EtBr) and visualized under UV on Gel Doc XR+ imaging system (BIO-RAD, München, Germany) to detect the presence or absence of gene specific bands.

### Library preparation and miRNA deep sequencing

MiRNA library preparation and miRNA deep sequencing was performed by a commercial company GATC BioTech AG (Konstanz, Germany) according to the Illumina small RNA sample preparation protocol. One μg of miRNA enriched total RNA samples from granulosa cells were subjected to construction of tagged miRNA sequencing libraries using TruSeq Small RNA Sample Prep Kit according to manufacturer’s instructions. Briefly; specific 3´and 5´ RNA adapters (S1 Table) were ligated to each end of the RNA template followed by purification of the 1^st^ and 2^nd^ adapter ligation products. The 3´ RNA adapter is modified in a way to capture miRNAs and other small RNA species in the sample. Single stranded cDNA was synthesized by reverse transcription using RT primers (S1 Table). cDNA samples were amplified by PCR using specific primers (S1 Table). PCR products were gel purified and band fraction size range of 140–160 nucleotides were excised using clean scalpel. Finally, single read clusters were generated and sequencing was performed on Illumina HiSeq 2000 in single read mode with read length of 50 bases. Base-calling, data filtering and index sorting were performed by the CASAVA Pipeline version 1.8.0. Raw FASTQ sequence reads of 50 nucleotides length were obtained.

### Sequence Quality control and pre-processing

FASTQ files were subjected to preliminary sequence quality control procedures using FASTQC version 0.10.0 (http://www.bioinformatics.babraham.ac.uk/projects/fastqc). Per base sequence quality and per sequence quality scores were thoroughly inspected. The 5´ adapter, 3´ adapter, RT primers, PCR primers and their corresponding reverse complementary sequences were trimmed. Moreover, sequence reads with Phred score lower than 18 and sequence reads shorter than 18 bp after trimming were removed from all the data sets using both Cutadapt [20] (https://code.google.com/p/cutadapt/) and Seqtk tools (https://github.com/lh3/seqtk). The raw FASTQ files and processed CSV files have been deposited in NCBI's Gene Expression Omnibus and are accessible through GEO Series accession number GSE56002.

### Sequence read alignment and detection of bovine miRNAs

Detection of both known and novel miRNAs was elucidated using miRDeep2.0.0.5 software package [21]. The *Bos taurus* genome release 72 was downloaded from ensemble genome browser (ftp://ftp.ensembl.org/pub/release-72/fasta/bos_taurus/dna/) and indexed with Bowtie 1.3 [22] (http://sourceforge.net/projects/bowtie-bio/files/bowtie2/2.1.0/). Moreover, the FASTA file of all matured bovine miRNA, precursor bovine miRNAs and miRNAs of other species (human, mouse and rat) were downloaded from miRBase database (release 20: June 2013) (http://mirbase.org/ftp.shtml). Sequence reads were mapped to bovine reference genome and aligned sequence reads were blasted against both mature and precursor miRNAs of bovine, human, mouse and rat.

### Prediction of novel miRNAs

Novel miRNAs and their respective read counts were inferred using miRDeep2 software package as described in [23]. MiRDeep2 predicts the probability of unannotated sequence being novel miRNA based on the genomic context which surrounds the sequence and the capability of the sequence to fold into hairpin structure with low free energy [21]. Secondary structure of miRNA precursor was predicted using RNAfold [24] and minimum free energy algorithm [25]

### Data normalization and differential expression of miRNAs

The workflow of miRDeep2 was followed to generate the expression data of all known miRNAs. Raw expression data was normalized as previously described [16]. Differences in the number of reads in each sample were normalized using DESeq2 by generating a “hypothetical reference” with read count equals to the geometric mean of read count of all samples. DESeq2 uses a negative binomial distribution model to count on the biological and technical variability among samples. Finally, the normalized read count of each miRNA in each sample was obtained by dividing the read count each miRNA to the geometric mean of all samples. Analysis of differentially expressed miRNAs was designed in a way to evaluate the differences in miRNA expression in granulosa cells derived from preovulatory dominant and their subordinate follicles counterparts. Differential expression of miRNAs was calculated from read count data using the DESseq2 of the R package as described [26]. MiRNAs with log_2_ fold change differences ≥ 1, *p*_-_value ≤ 0.05 and false discovery rate (FDR) ≤ 0.1 were considered as statistically significantly differentially expressed. PermutMatrix was used for clustering analysis and heat map generation of differentially expressed miRNAs [27]

### MiRNA target gene prediction and functional annotation

Interaction between differentially expressed miRNAs and their target mRNA was predicted using miRecords; a widely used web-based database to predict animal miRNA-target mRNA interactions [28]. Target genes that were predicted by at least 4 target prediction algorithms within miRecords were filtered for further analysis. For miRNAs whose target genes were not available in miRecords were searched in miRDB [29] and targetscan cow release 6.2 [30]. Following this, the list of predicted target genes of individual miRNAs were imported to DAVID Bioinformatics systems; a freely available bioinformatics tool (http://david.abcc.ncifcrf.gov/). Gene ontology (GO) and canonical pathways significantly enriched by the predicted target genes of each miRNAs were identified. Canonical pathways were identified from the Kyoto Encyclopaedia of Genes and Genomes (KEGG) database [31].

### Validation of candidate miRNAs using qPCR

Nine differentially expressed candidate miRNAs identified by NGS (bta-miR-132, bta-miR-212, bta-miR-21-3p, bta-miR-96, bta-miR-182, bta-miR-221, bta-miR-335, bta-miR-708 and bta-miR-214) were randomly selected for quantitative real time PCR (qPCR) validation. Furthermore, the relative abundance of these candidate miRNAs was assessed in theca cells, COC and follicular fluid. For this, first strand cDNA was synthesized from equal amount of total RNA input using miRCURY LAN Universal cDNA synthesis kit (Exiqon, Vedbaek, Denmark) according to the manufacturer´s instruction. cDNA templates were 40X diluted using nuclease free water. 4 μl of diluted cDNA template was mixed with 5 μl of ExiLENT SYBR Green Master mix and 1 μl of PCR primer mix (Exiqon, Vedbaek, Denmark). QPCR amplification was performed in a StepOnePlus Real-Time PCR systems (Applied Biosystems, Foster City, CA, USA) with the thermocycling conditions of initial heating at 95°C for 10 minute followed by 40 cycles of amplification step at 95°C for 10 s and 60°C for 1 min. Melting curve analysis was performed to assess specific amplification of primers.

### Plasmid Construction

To experimentally validate the Insilco target gene prediction of differentially expressed miRNAs, we selected the miR-183 cluster miRNAs (bta-miR-183, bta-miR-182 and bta-miR-96); which are predicted to target the Forkhead box protein O1 (*FOXO1*) gene. For this we constructed plasmid DNA containing part of the 3′-UTR of bovine *FOXO1* gene harbouring the putative miRNA binding sites for bta-miR-183, bta-miR-182 and bta-miR-96. The 3´-UTR DNA fragment was amplified from genomic DNA of bovine granulosa cell using specific primers (S1 Table). Simultaneously, mutant *FOXO1* 3´-UTR constructs with mutations on target recognition sites of miR-183, miR-182 and miR-96 were generated. The wild-type and mutant *FOXO1* 3´-UTR were cloned between the *SacI/XhoI* restriction sites downstream of the pmirGLO Dual-Luciferase miRNA Target Expression Vector (Promega, Madison, WI, USA). Presence of miRNA binding sites in the plasmid constructs were confirmed by sequencing before and after cloning.

### Luciferase reporter assay

Primary granulosa cells were cultured in 24-well plate as previously described [32]. Sub-confluent (70–80% confluent), cultured cells were co-transfected with *FOXO1* wild-type or mutant 3´-UTR reporter constructs and bta-miR-183/182/96 miRNA mimics using lipofectamine 2000 (Invitrogen, Darmstadt, Germany). Cells were lysed 24 hours post transfection and activity of firefly and Renilla Luciferase were determined using Dual-Glo luciferase assay kit (Promega, Madison, WI, USA). Data was calculated as the ratio of Firefly luciferase to Renilla activity.

### Quantification of genes targeted by miR-183 cluster using qPCR

Primers of the predicted target genes; *FOXO1* was designed from the reference mRNA sequence using primer3 program (S1 Table). Total RNA isolated from granulosa cells of preovulatory dominant and subordinate follicles was used to synthesize cDNA as described above. Then, a PCR master mix comprising of 7.4 μl ddH2O, 0.3 μl of forward primer, 0.3 μl of reverse primer and 10 μl of 1x SYBR Green I master mix (Bio-Rad) was mixed with 2 μl of cDNA template to make a reaction volume of 20 μl. Finally, thermo cycling conditions were set to 3 min at 95°C followed by 40 cycles of 15 seconds at 95°C and 1 min at 60°C. Melting curve analysis was performed to assess specific amplification of primers.

### Statistical analysis

Expression data of selected candidate miRNAs generated by qPCR in granulosa cells, theca cells, cumulus-oocyte-complex and follicular fluid derived from preovulatory dominant and subordinate follicles was analyzed using the comparative threshold cycle (Ct) method [33]. Expression data was normalized against the geometric mean of the expression of 2 endogenous reference miRNAs; 5*s* Ribosomal RNA (*5s* rRNA) and *U6* small non-coding small nuclear RNA (*U6* snRNA). Similarly, expression of target mRNA in granulosa cells of preovulatory dominant and subordinate was analysed using the comparative threshold cycle (Ct) method [33]. Expression data of target mRNA was normalized against the expression of *GAPDH*. Two-tailed student´s *t*-test was performed to discover statistical differences in the mean expression value between treatment groups and statistical significance was defined at *p*-value ≤ 0.05. GraphPad prism 5 (GraphPad, San Diego, CA) was used to plot the relative expression of selected miRNAs and mRNA. QPCR was performed in biological triplicates and relative expression values are presented as mean ± SD of normalized Ct values.

## Results

A total of 5 preovulatory dominant and 76 subordinate follicles were obtained from 5 experimental animals. The mean surface diameter of retrieved preovulatory dominant follicles (15.4 ± 3.68 mm) was significantly different (*p* < 0.001) compared to the mean surface diameter of subordinate follicles (6.53 ± 0.99 mm). Moreover, analysis on the purity of isolated granulosa cells from these two follicular categories showed strong presence of granulosa cell specific marker gene (*FSHR*) and a very negligible level of theca cell specific marker gene (*CYP17A1*) in all samples (S1 Fig).

### Characterization of miRNA deep sequencing data

To investigate the involvement of miRNAs in bovine follicular development specifically during the preovulatory stage, 6 miRNA sequencing libraries were generated using granulosa cells derived from preovulatory dominant and subordinate follicles. Libraries were sequenced using the Illumina HiSeq2000 small RNA deep sequencing technology and 50 bases long sequence reads were generated. Accordingly, 8.2 and 8.9 million reads were obtained from libraries of the preovulatory dominant and subordinate follicles, respectively. After filtering low-quality reads and empty adaptors, the mean quality read of the biological triplicates was 2.4 and ~3 millions in preovulatory dominant and subordinate follicle libraries, respectively. Quality filtered sequence reads were used for detection of known annotated and prediction of novel miRNAs. From all reads which passed the quality control criteria, 663,338 reads in preovulatory dominant and 928,373 in subordinate follicles were mapped to the bovine reference genome, comprising 27.6 and 31.4% of the total quality reads obtained, respectively. Furthermore, 343,221 reads in preovulatory dominant and 467,028 in subordinate follicles were found to be similar with known bovine miRNAs reported in miRBase release 20 (Table 1).

**Table 1:** Summary of sequence reads alignment to bovine reference genome and known miRNAs annotated in miRBase.

Group	SampleID^*^	Total number of QC reads	Number of mapped reads ^€^	Mapped reads(%)	Reads Aligned to known miRNAs	Aligned to Known miRNAs ^£^(%)
Dominant follicle	D1	1,925,662	599,377	31.1	345,689	57.7
	D2	1,967,061	392,924	20.0	255, 260	65.0
	D3	3,161,472	997,715	31.6	428,716	43.0
Subordinate follicle	S1	3,069,606	861,596	28.1	459,794	53.4
	S2	2,895,393	939,835	32.5	520,377	55.4
	S3	2,922,775	983,688	33.7	420,913	42.8

^*^: D1, D2, D3 denote for biological triplicates of preovulatory dominant follicles and S1, S2, S3 denote for biological triplicates of subordinate follicles

^**€**^: Number of quality filtered reads aligned to bovine reference genome release 72.

^**£**^: Proportion of mapped sequence reads aligned to known annotated miRNAs in miRBase release 20

### MiRNAs expressed in Granulosa cells of preovulatory dominant and subordinate follicles

MiRNAs with as least 1 read count in at least two of the three biological replicates were considered as detected. Accordingly, a total of 315 and 323 known bovine miRNAs were detected in preovulatory dominant and subordinate follicles, respectively of which 287 miRNAs were commonly detected in both sample groups. However, 28 miRNAs including bta-miR-96 and bta-miR-122 were found to be specific to preovulatory dominant. While, 36 miRNAs including bta-miR-409a and bta-miR-449b were unique to subordinate follicles.

The abundance of detected miRNAs showed a broader range both in the preovulatory dominant and subordinate follicles. Among which, bta-miR-26a and bta-miR-10b were the two most abundantly expressed miRNAs with a read count of 49000 and 28169 in preovulatory dominant and 77,730 and 62,390 in subordinate follicles; accounting for 22.5 and 30% of the sequence reads aligned to known miRNAs, respectively. Similarly, three isoforms of the let-7 family (bta-let-7a-5p, bta-let-7f and bta-let-7i) comprised 8.5 and 7.3% of sequence reads aligned to known miRNAs in preovulatory dominant and in subordinate follicles, respectively. Nevertheless, the overwhelming majority of the detected miRNAs in both libraries had less than 50 read counts (S2 Fig). Among the top 10 abundantly expressed miRNAs in each group, 7 miRNAs (bta-miR-26a, bta-miR-10b, bta-let-7a-5p, bta-let-7f, bta-let-7i, bta-miR-27b and bta-miR-191) were commonly expressed in both preovulatory and subordinate follicles (Table 2). List of all detected miRNAs with their normalized read count is indicated in (S2 Table).

**Table 2:** List of Top 10 highly abundantly expressed miRNAs in granulosa cells of preovulatory dominant and subordinate follicles.

Preovulatory dominant follicle		Subordinate follicle	
miRNA ID	Average read count ^§^	miRNA ID	Average read count^§^
bta-miR-26a	48999.00	bta-miR-26a	77730.33
bta-miR-10b	28168.67	bta-miR-10b	62390.00
bta-miR-202	12209.00	bta-miR-92a	13653.33
bta-let-7a-5p	10838.33	bta-let-7f	13331.00
bta-let-7f	9595.33	bta-miR-27b	13194.67
bta-miR-22-3p	8710.33	bta-miR-99b	12241.33
bta-let-7i	8695.67	bta-let-7a-5p	11003.67
bta-miR-21-5p	8695.33	bta-let-7i	9734.67
bta-miR-27b	8476.67	bta-miR-191	8563.00
bta-miR-191	7700.33	bta-miR-143	8397.67

^§:^ The arithmetic mean of read counts of biological triplicates

### Expression pattern of miR-#-5p and miR-#- 3p arms

We found that majority of the detected miRNAs to be derived from the miR-#-5p arm of miRNA precursor. In our sequencing libraries, there were 36 and 39 miRNAs derived from the miR-#-3p arm of the miRNA precursor in preovulatory dominant and subordinate follicles, respectively. Interestingly, 26 miR-#-5p/3p duplexes were detected, among them 22 duplexes were commonly expressed in both the dominant and subordinate follicles (Table 3). The expression of majority of the miR-#-3p arms is lower than their corresponding miR-#-5p arm. For instance, the expression of the 3p arm of bta-let-7a in dominant and subordinate follicles was 27 and 34 reads, respectively. Whereas, the expression of its corresponding 5p arm in dominant and subordinate follicles was 10,838 and 11,004 reads, respectively. MiRNAs like miR-151-5p and miR-126-5p exhibited similar level of expression as their corresponding 3p arms. However, the expression level of miR-22-3p both in dominant and subordinate follicles was higher than miR-22-5p. Moreover, miR-199a-5p/3p and miR-2313-5p/3p duplexes were exclusively expressed in preovulatory dominant follicles and absent in subordinate follicles. A representative miRNA precursor (bta-mir-126) with functional 5p and 3p arms is shown in S3 Fig.

**Table 3:** Expression pattern of miRNA-duplexes in preovulatory dominant and subordinate follicle libraries.

miRNA-Duplex^*^	Read count in Preovulatory Dominant follicles		Read count in Subordinate follicles	
	miR-#-5p	miR-#-3p	miR-#-5p	miR-#-3p
bta-let-7a	10838	27	11004	34
bta-mir-21	8695	399	4662	120
bta-mir-151	1465	1457	1971	2187
bta-mir-99a	687	12	1981	13
bta-mir-423	654	388	1081	806
bta-mir-424	460	69	87	12
bta-mir-6119	395	7	1015	20
bta-mir-425	164	9	143	10
bta-mir-199a	123	244	0	0
bta-mir-129	83	60	3	1
bta-mir-17	50	13	157	15
bta-mir-2483	35	7	17	10
bta-mir-126	27	23	74	56
bta-mir-22	20	8710	2	1792
bta-mir-1388	17	1	29	3
bta-mir-362	11	2	15	1
bta-mir-345	6	30	3	15
bta-mir-2313	5	118	0	0
bta-mir-503	5	8	4	4
bta-mir-193a	2	5	2	4
bta-mir-2320	2	2	2	1
bta-mir-2284t	1	5	3	14
bta-mir-411c	1	2	1	1
bta-mir-545	1	1	1	1
bta-mir-455	0	0	2	8
bta-mir-9	0	0	2	1

*: miRNA-duplex is the 5p and 3p arm of a single miRNA precursor

### Differentially expressed miRNAs in Granulosa cells of preovulatory dominant follicles

**Fig 1:**
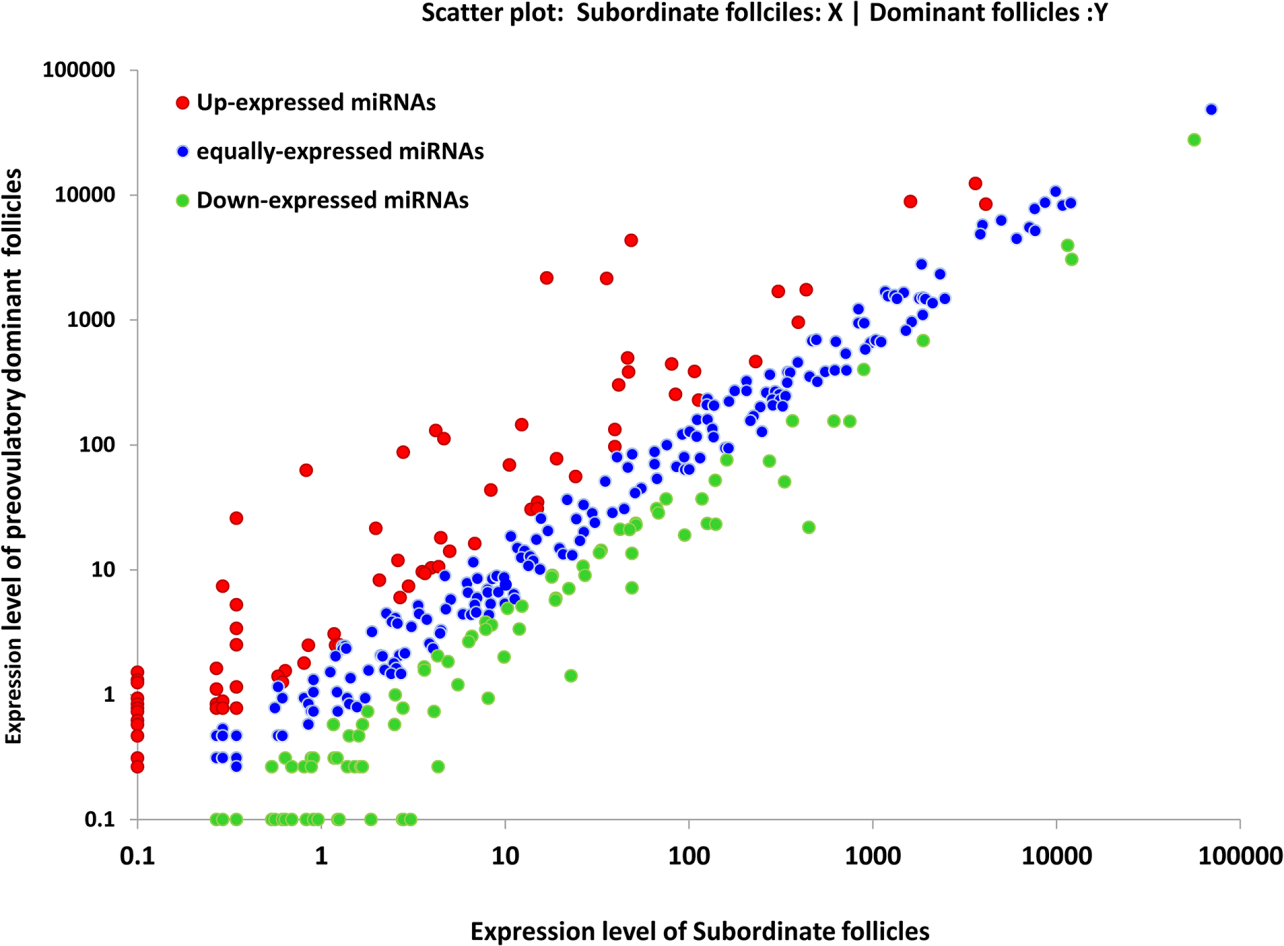
Scatter plot of miRNA expression level in preovulatory dominant and subordinate follicles. The expression level of preovulatory dominant and subordinate follicles is indicated in Y and X axis, respectively. Up-expressed miRNAs; with ratio ≥ 2 are labeled with red points. Equally expressed miRNAs; with 0.5 < ratio ≤ 2 are labeled with blue points. Down-expressed miRNAs; with ratio ≤ 0.5 are labeled with green points. Ratio = Normalized expression level in preovulatory dominant follicles / Normalized expression level in subordinate follicles

**Fig 2:**
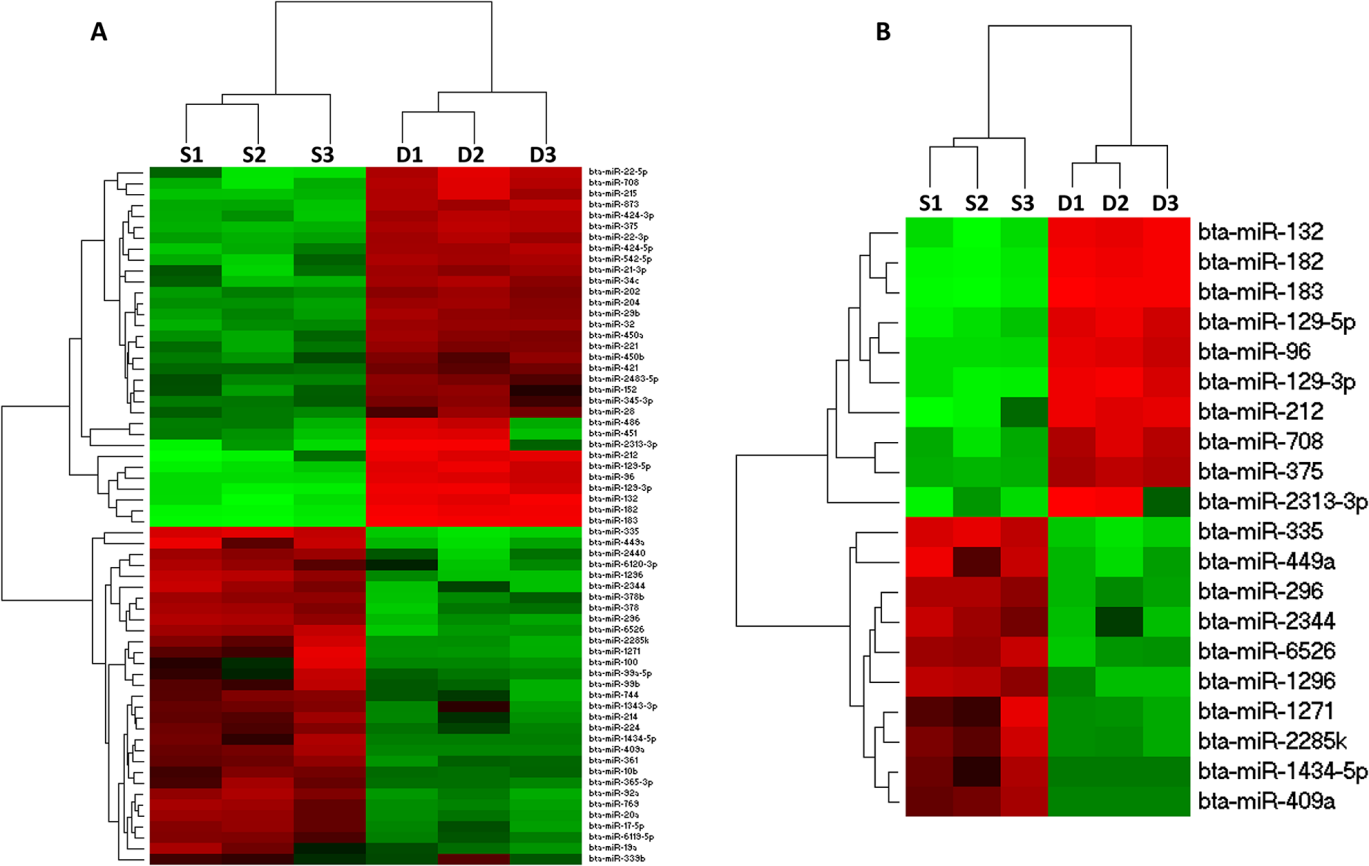
Hierarchical clustering of differentially expressed miRNAs in granulosa cells of preovulatory dominant and subordinate follicles. Heat map of all differentially expressed miRNAs in preovulatory dominant follicles (**A**) and 20 top miRNAs differentially expressed in preovulatory dominant follicles (**B**). Red and green blocks represent up and down regulated miRNAs, respectively. Legend: S1-S3 subordinate follicle triplicates and D1-D3 preovulatory dominant follicle triplicates.

Comparison of the level of expression of all detected miRNAs was determined by calculating the ratio of the normalized expression values of each miRNA in both follicles. It was observed that majority of the detected miRNAs are equally expressed in both preovulatory and subordinate follicles (Fig 1). Differential expression analysis revealed 64 miRNAs to be significantly differentially expressed between preovulatory dominant and subordinate follicles. In comparison with subordinate follicles, 34 matured miRNAs including miR-132 cluster (bta-miR-132 and bta-miR-212) and miR-183 cluster (bta-miR-183, bta-miR-182 and bta-miR-96) were significantly enriched in preovulatory dominant follicles (Table 4). While, the expression level of 30 other matured miRNAs including bta-miR-409a, bta-miR-335, bta-miR-378 and bta-miR-17-5p were significantly reduced in preovulatory dominant follicles (Table 5). The Log_2_ fold change values in preovulatory dominant follicles range from 7.03 (bta-miR-183) up to -16.4 (bta-miR-409a). The hierarchical clustering of all and top 20 differentially expressed miRNAs in granulosa cells derived from the two follicular categories is described in Fig 2. Majority of the differentially expressed miRNAs were detected in both follicular categories. However, 2 miRNAs (bta-miR-96 and bta-miR-375) enriched in preovulatory dominant follicles were uniquely detected in granulosa cells of preovulatory dominant follicles. Similarly, 3 miRNAs (bta-miR-1434, bta-miR-4099 and bta-miR-2344) were detected only in granulosa cells of subordinate follicles.

**Table 4:** List of miRNAs up-regulated in granulosa cells of preovulatory dominant follicles.

miRNAs ID	Fold change	*p*-value	FDR
bta-miR-183	7.03	< 0.0001	< 0.0001
bta-miR-182	6.49	< 0.0001	< 0.0001
bta-miR-96	6.42	< 0.0001	< 0.0001
bta-miR-129-3p	6.13	< 0.0001	< 0.0001
bta-miR-132	5.91	< 0.0001	< 0.0001
bta-miR-129-5p	5.00	< 0.0001	< 0.0001
bta-miR-2313-3p	4.96	< 0.0001	< 0.0001
bta-miR-212	4.67	< 0.0001	< 0.0001
bta-miR-375	4.62	0.0007	0.0073
bta-miR-708	3.56	< 0.0001	< 0.0001
bta-miR-215	3.42	< 0.0001	< 0.0001
bta-miR-22-5p	3.37	< 0.0001	0.0005
bta-miR-451	3.03	0.0003	0.0034
bta-miR-873	2.86	< 0.0001	< 0.0001
bta-miR-424-3p	2.69	< 0.0001	< 0.0001
bta-miR-424-5p	2.47	< 0.0001	< 0.0001
bta-miR-486	2.46	0.0006	0.0061
bta-miR-22-3p	2.46	< 0.0001	< 0.0001
bta-miR-542-5p	2.40	< 0.0001	< 0.0001
bta-miR-34c	2.15	0.0057	0.0422
bta-miR-29b	2.06	0.0011	0.0099
bta-miR-32	2.03	< 0.0001	< 0.0001
bta-miR-204	2.00	< 0.0001	< 0.0001
bta-miR-21-3p	1.85	< 0.0001	0.0006
bta-miR-202	1.77	< 0.0001	< 0.0001
bta-miR-450a	1.75	< 0.0001	< 0.0001
bta-miR-221	1.58	< 0.0001	< 0.0001
bta-miR-450b	1.28	< 0.0001	0.0002
bta-miR-28	1.27	0.0003	0.0028
bta-miR-152	1.20	0.0052	0.0393
bta-miR-2483-5p	1.19	0.0063	0.0457
bta-miR-345-3p	1.13	0.0125	0.0760
bta-miR-339b	1.02	0.0005	0.0056
bta-miR-421	1.01	< 0.0001	< 0.0001

Fold change values are in Log_2_ scale

**Table 5:** List of miRNAs down-regulated in granulosa cells of preovulatory dominant follicles.

miRNAs ID	FC	*p*-value	FDR
bta-miR-409a	-16.43	0.0097	0.0642
bta-miR-1434-5p	-16.39	0.0143	0.0847
bta-miR-335	-4.35	< 0.0001	< 0.0001
bta-miR-449a	-3.88	0.0001	0.0011
bta-miR-2344	-3.48	0.0073	0.0512
bta-miR-1296	-2.78	< 0.0001	< 0.0001
bta-miR-1271	-2.72	< 0.0001	0.0009
bta-miR-6526	-2.60	< 0.0001	< 0.0001
bta-miR-296	-2.45	< 0.0001	< 0.0001
bta-miR-2285k	-2.33	< 0.0001	0.0003
bta-miR-100	-2.29	0.0007	0.0073
bta-miR-378	-2.01	< 0.0001	< 0.0001
bta-miR-92a	-1.99	< 0.0001	< 0.0001
bta-miR-769	-1.90	< 0.0001	< 0.0001
bta-miR-378b	-1.85	< 0.0001	0.0005
bta-miR-2440	-1.77	0.0160	0.0939
bta-miR-20a	-1.70	< 0.0001	< 0.0001
bta-miR-6120-3p	-1.64	0.0114	0.0713
bta-miR-99b	-1.55	0.0011	0.0106
bta-miR-99a-5p	-1.47	0.0027	0.0217
bta-miR-17-5p	-1.45	< 0.0001	0.0003
bta-miR-365-3p	-1.33	0.0108	0.0682
bta-miR-19a	-1.27	0.0166	0.0956
bta-miR-214	-1.25	0.0106	0.0682
bta-miR-361	-1.24	< 0.0001	0.0001
bta-miR-744	-1.21	0.0057	0.0422
bta-miR-6119-5p	-1.17	< 0.0001	< 0.0001
bta-miR-1343-3p	-1.11	0.0024	0.0206
bta-miR-224	-1.05	0.0017	0.0153
bta-miR-10b	-1.03	< 0.0001	< 0.0001

Fold change values are in Log_2_ scale

### Prediction of novel bovine miRNAs

**Fig 3:**
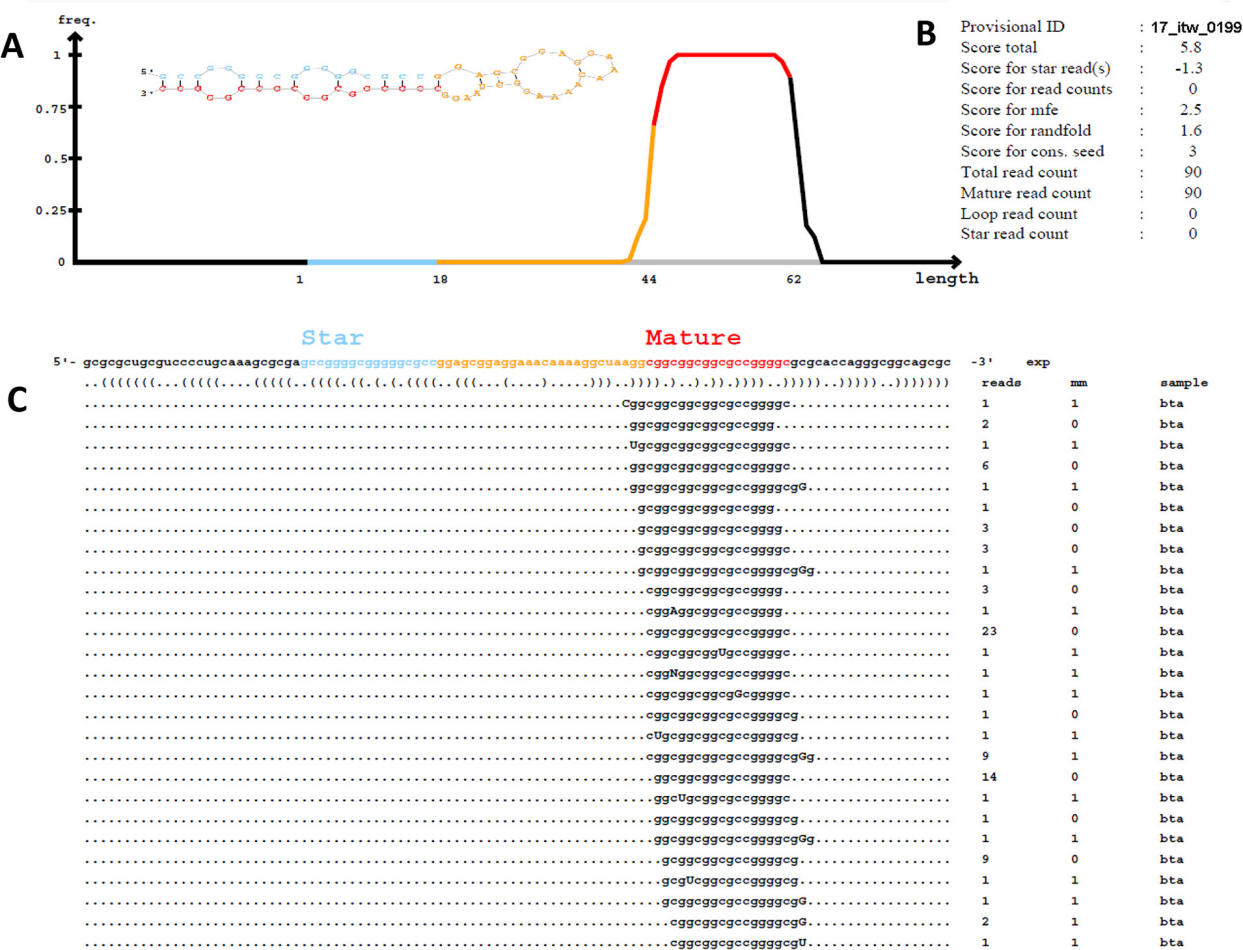
Graphic illustration of a representative predicted novel miRNA by miRDeep2. The primary miRNA hairpin with both mature and star miRNAs highlighted with red and blue colors, respectively (**A**). MiRDeep2 scores and provisional ID are shown (**B**). The consensus matured miRNA sequence and other isomiRs and their corresponding read counts are indicated. Mismatched nucleotides of isomiRs with the miRNAs hairpin are written in capital letter (**C**).

Novel miRNAs detected in at least one of the three biological replicates with at least 1 read count are reported. A total of 11 novel miRNAs were predicted by miRDeep2 software, of which 7 were commonly predicted in both the preovulatory dominant and subordinate follicles. However, 3 and 1 predicted novel miRNAs were unique for preovulatory dominant and subordinate follicles, respectively (Table 6). Sequence homology search was made for all the predicted novel miRNAs using the basic local alignment search tool (BLASTN) application against all known annotated matured miRNAs in miRBase release 20. It was shown that no known homologous miRNAs was aligned to all the predicted novel miRNAs. Genomic context analysis of the predicted novel miRNAs revealed that 6 novel miRNAs were transcribed from intergenic region, 4 from intronic region of transcripts (*PDCL2*, *AFF1*, *RGS22 and TEX14*) and 1 other from exonic regions of the *MAGED1* gene (Table 6). A representative readout of a predicted novel miRNAs is shown in Fig 3.

**Table 6:** Predicted novel miRNAs expressed in granulosa cells of preovulatory dominant and/or subordinate follicles.

Provisional miRNA ID	Matured miRNA Sequence	genomic coordinates and strand of miRNA precursors	Average read count in DF	Average read count in SF	Genomic region of novel miRNAs and overlapping transcript
4_itw_0216	gggcgcgcgccgcggcu	4:89824112..89824190:-	-	323	Intergenic
X_itw_0135	ccggggccgcgguuccgc	X:62080875..62080910:-	1282	-	Intergenic
X_itw_0174	cccgugaucuggccaaaccc	X:95711630..95711673:-	17.5	-	Exonic, MAGED 1
6_itw_0271	caaaaaguucguccagauuuuu	6:72695823..72695883:+	13.5	-	Intronic, PDCL 2
9_itw_0214	cccgcggggcgcgcgccug	9:25842847..25842909:-	314	2580.3	Intergenic
4_itw_0009	gguggcggggggagguc	4:114121560..114121624:-	149	93.5	Intergenic
16_itw_0200	cggcggcggcgccggggc	16:26800409..26800490:+	62	26.5	Intergenic
17_itw_0199	cggcggcggcgccggggcgcg	17:72247500..72247567:+	51.5	46.5	Intergenic
6_itw_0083	uaaaaguuugguuggguuuuu	6:103797778..103797838:+	20.7	18	Intronic, AFF1
14_itw_0227	gggggggggccggggcc	14:66538774..66538818:+	7.5	20.5	Intronic, RGS22
19_itw_0090	ggagaggacaccgucugagugg	19:9830946..9830993:-	12	73	Intronic, TEX14

### Target gene prediction, Gene ontology and pathways enriched by differentially expressed miRNAs.

**Fig 4:**
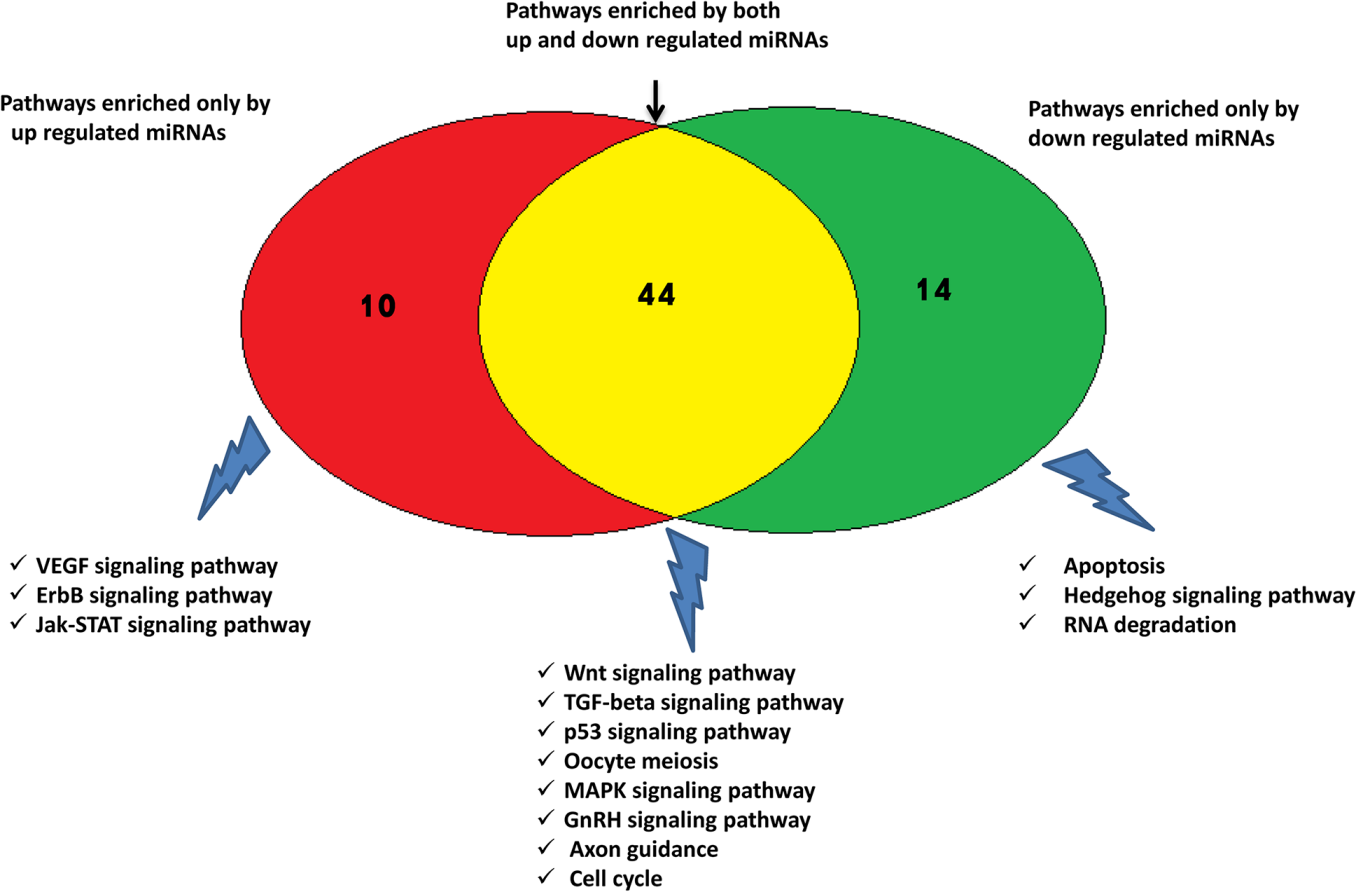
Venn diagram showing number of canonical pathways enriched by predicted target genes of differentially expressed miRNAs. Representative pathways enriched by predicted target of only up regulated miRNAs (red), only down regulated miRNAs (green) and both up and down regulated miRNAs (yellow) are shown.

To understand the functional involvement of differentially expressed miRNAs in bovine follicular development, target genes of each differentially expressed miRNAs were predicted and used to determine the most significantly enriched canonical pathways. Gene ontology (GO) analysis on predicted target genes of differentially expressed miRNAs revealed that, biological processes associated with transcription regulation, regulation of cell proliferation and cell death were among the highly enriched GO terms. Representative GO terms enriched by predicted target genes are indicated in (S3 Table). Apart from this, a total of 64 canonical pathways were enriched by the predicted target genes of differentially expressed miRNAs. Pathways important in oncogenesis (pathways in cancer and endometrial cancer), cell adhesion (Axon guidance, Focal adhesion and Gap junctions), cell proliferation (MAPK signaling pathway, Wnt signaling pathway, and cell-cycle), cell survival (TGFβ signaling pathway) and metabolism (GnRH and insulin signaling pathway) were among the pathways enriched by both up and down regulated miRNAs. Pathways like VEGF signalling pathway, ErbB signaling pathways and Jak-STAT signaling pathway were enriched only by miRNAs up regulated in preovulatory dominant follicles. Interestingly, apoptosis pathway, RNA degradation pathway and Hedgehog signaling pathway were enriched only by down regulated miRNAs in preovulatory follicles (Fig 4). Representative list of pathways known to be involved in ovarian follicular development along with the list of miRNAs predicted to modulate are indicated in S4 Table.

### Validation of candidate miRNAs using qPCR

**Fig 5:**
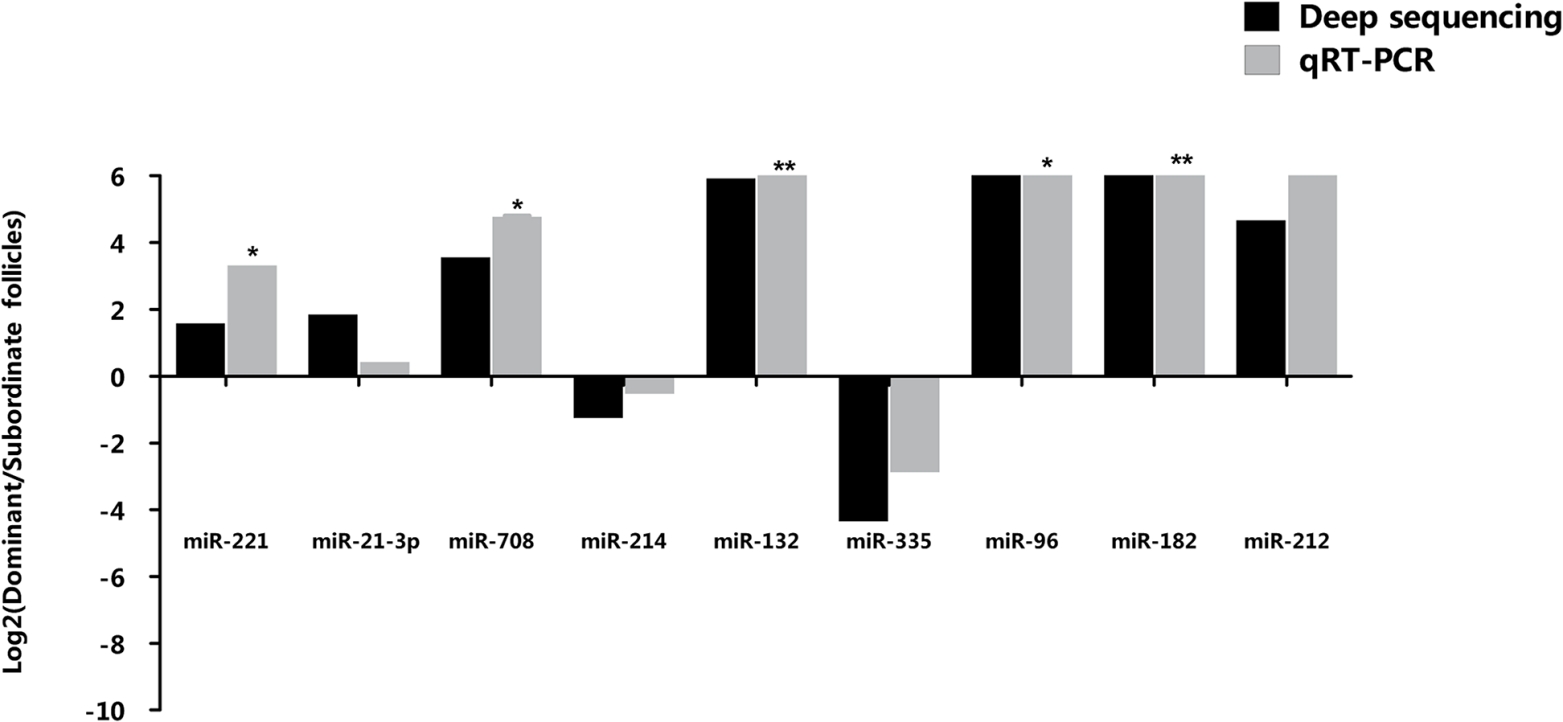
QPCR validation of selected candidate miRNAs differentially expressed between preovulatory dominant and subordinate follicles. The relative abundance of individual miRNAs is presented as the ratio of preovulatory dominant and subordinate follicles. The mean expression value of target miRNA was normalized against the expression of endogenous control U6 snRNA and 5s rRNAs. The normalized miRNA expression (2^-ΔCT^) in preovulatory dominant follicle was divided by the normalized miRNA expression in subordinate follicle and Log_2_ transformed. MiRNAs with Log_2_ ratio > 0 are up-regulated in preovulatory dominant follicles and miRNAs with Log_2_ ratio < 0 are down-regulated in preovulatory dominant follicles compared to the subordinate follicles counterparts. Statistical significance of each miRNA are represented by asterisks; *, *p*<0.05 and **, *p*<0.01.

Nine representative differentially expressed miRNAs were randomly selected to validate their expression in granulosa cells of preovulatory dominant and subordinate follicles using qPCR. As shown in Fig 5, the qPCR result was in agreement with the Illumina deep sequencing result.

### Expression of candidate miRNAs in Theca cell, COC and Follicular Fluid

**Fig 6:**
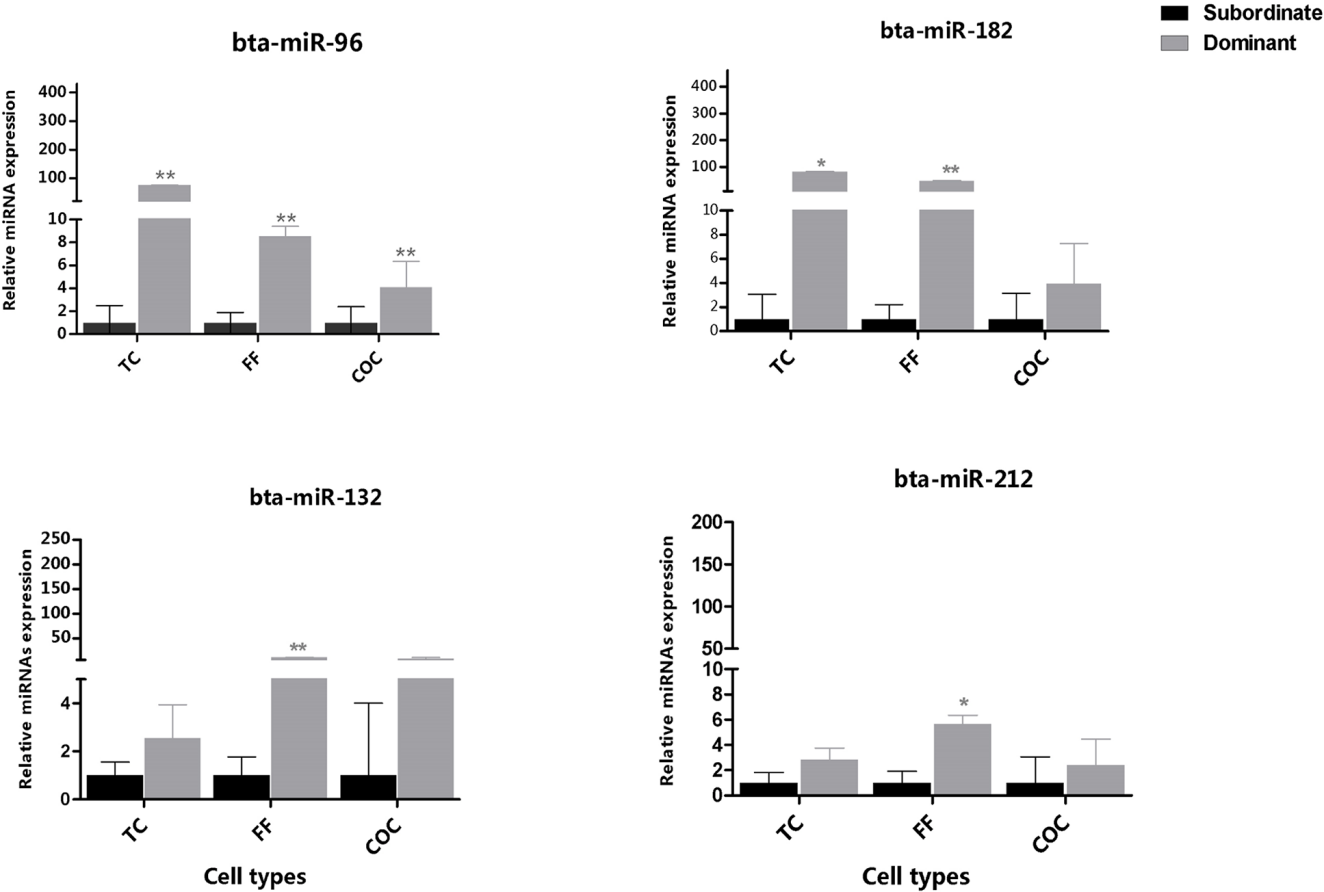
Expression pattern of members of the miR-183 cluster (bta-miR-182 and bta-miR-96) and miR-132 cluster (bta-miR-132 and bta-miR-212) in companion follicular cells of both preovulatory dominant and subordinate follicles using qPCR. The mean expression value of target miRNA was normalized against the expression of endogenous control U6 snRNA and 5s rRNAs. Relative expression values were calculated using ΔΔCT method. Error bars represent SD of ΔΔCT values. Statistical significance of each miRNA are represented by asterisks; *, *p*<0.05, **, *p*<0.01 and ***, *p*<0.001. Legend: TC = Theca cells COC = cumulus-oocyte-complex FF = follicular fluid.

**Fig 7:**
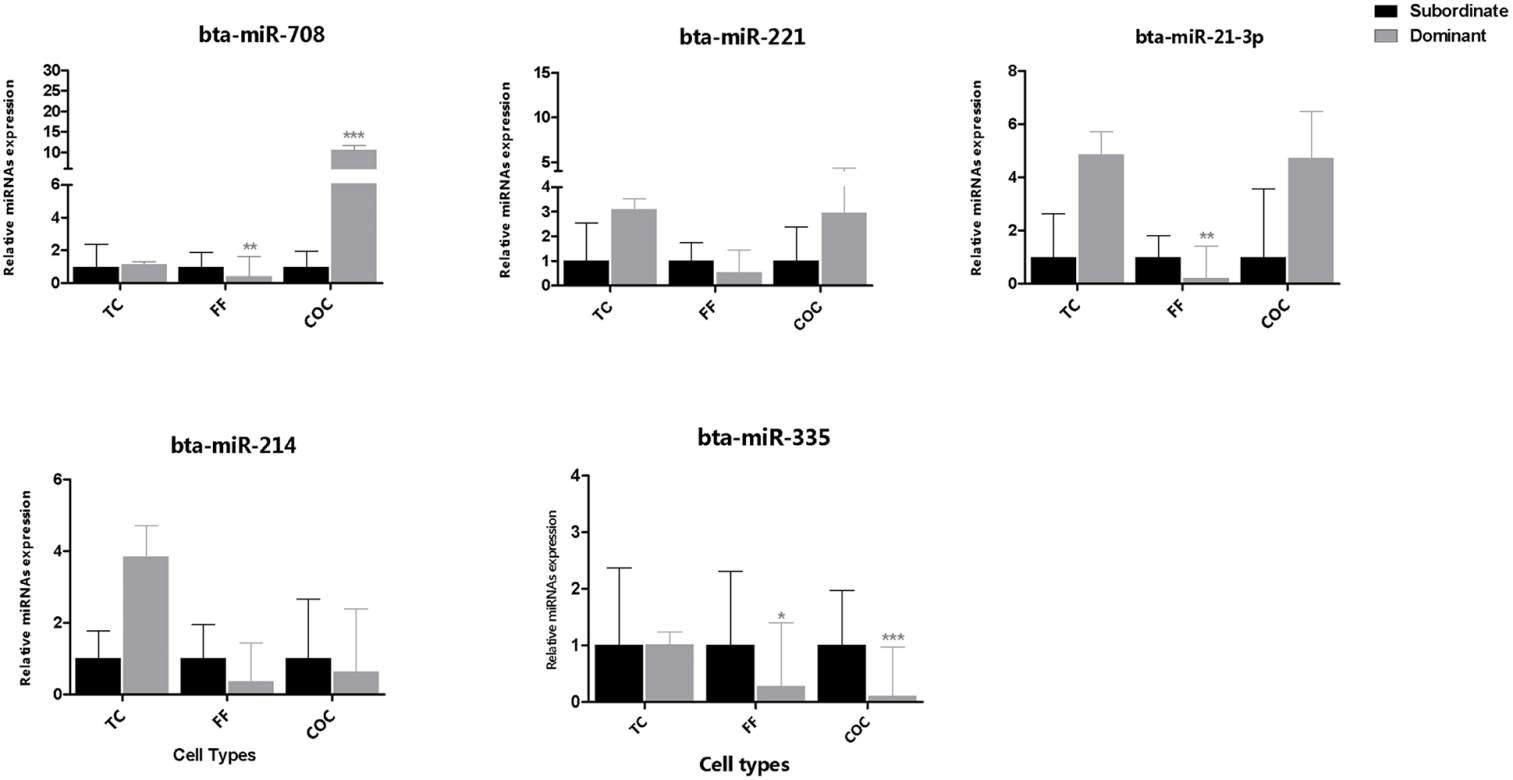
Expression pattern of bta-miR-708, bta-miR-221, bta-miR-21-3p, bta-miR-214 and bta-miR-335 in companion follicular cells of both preovulatory dominant and subordinate follicles using qPCR. The mean expression value of target miRNA was normalized against the expression of endogenous control U6 snRNA and 5s rRNAs. Relative expression values were calculated using ΔΔCT method. Error bars represent SD of ΔΔCT values. Statistical significance of each miRNA are represented by asterisks; *, *p*<0.05, **, *p*<0.01 and ***, *p*<0.001. Legend: TC = Theca cells COC = cumulus-oocyte-complex FF = follicular fluid.

The relative abundance of selected candidate miRNAs was determined in theca cells, COC and follicular fluid derived from preovulatory dominant and subordinate follicles where the corresponding granulosa cells were used for deep sequencing. Results showed that the relative abundance of miR-132 cluster (bta-miR-132 and bta-miR-212) and member of the miR-183 cluster (bta-miR-182, and bta-miR-96) was higher in theca cells, COC and follicular fluid of the preovulatory dominant follicles compared to the subordinate follicles counterparts (Fig 6). On the contrary, the relative abundance of bta-miR-335 was higher in theca cells, COC and follicular fluid of subordinate follicles compared to the preovulatory dominant follicle. Moreover, the follicular fluid of subordinate follicles was highly enriched with bta-miR-708, bta-miR-221, bta-miR-21-3p, bta-miR-335 and bta-miR-214 compared to the follicular fluid derived from dominant follicles (Figs 6 and 7).

### Validation of gene targeted by candidate miRNAs

**Fig 8:**
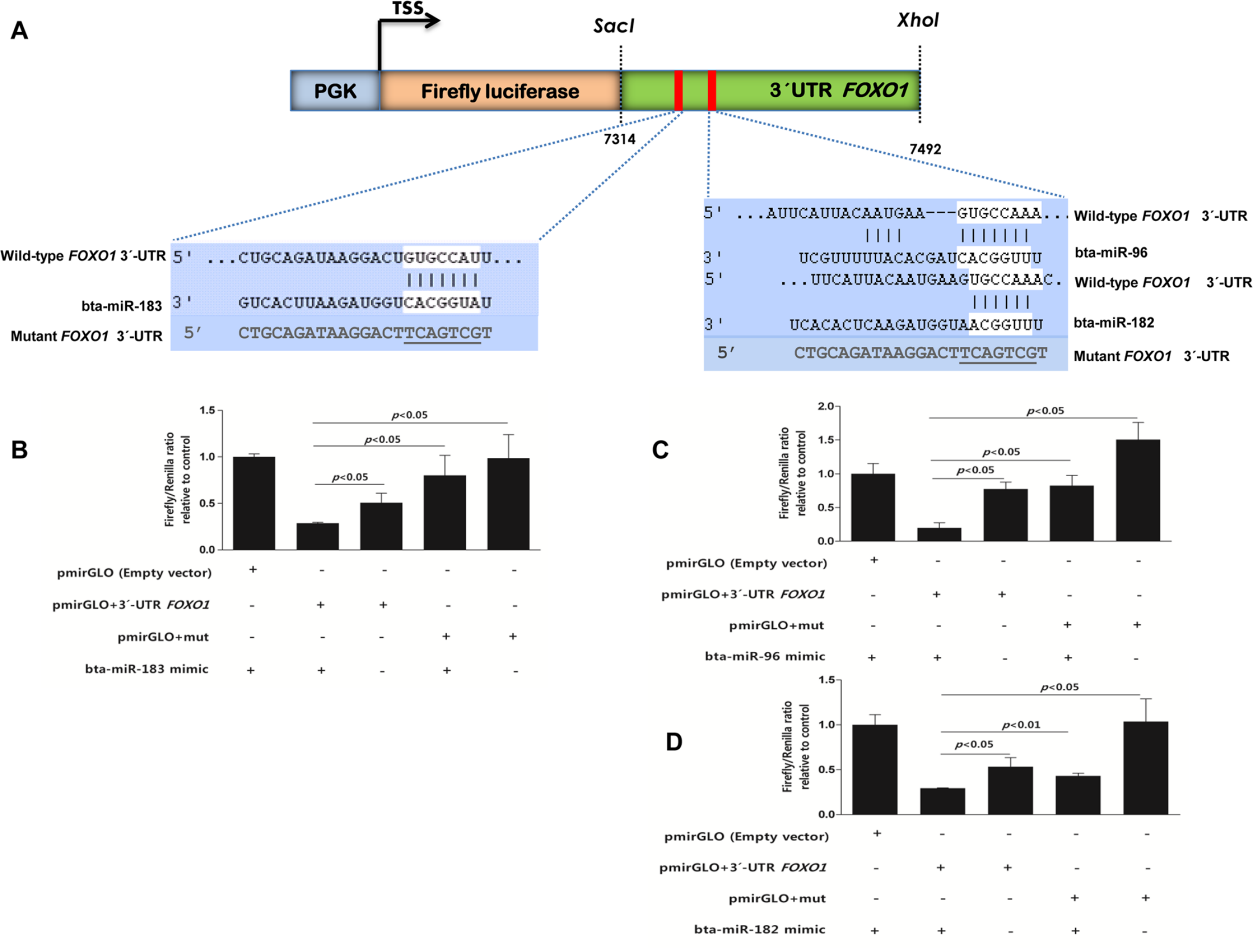
MiR-183 cluster miRNAs coordinately target *FOXO1* mRNA in bovine granulosa cells. Putative miR-183 cluster binding sites and their genomic coordinates in the 3´-UTR of bovine *FOXO1* mRNA were designed. Plasmid with wild-type and mutant sequences (underlined) for miRNA binding were fused downstream the firefly luciferase gene between *SacI* and *XhoI* restriction sites. The PGK promoter and transcription start site (TSS) of are indicated (A). Granulosa cells were transfected with or without bta-miR-183 mimic (B), with or without bta-miR-96 mimic (C), with or without bta-miR-182 mimic (D). The activity of luciferase was significantly inhibited when the bovine *FOXO1* 3´-UTR with wild type of miRNA binding sites was co-transfected with all miR-183 cluster miRNA mimics. However, the activity of Luciferase was not affected when *FOXO1* 3´-UTR with mutant sequences at the miRNA binding sites were co-transfected with or without miR-183 cluster miRNA mimics.

**Fig 9:**
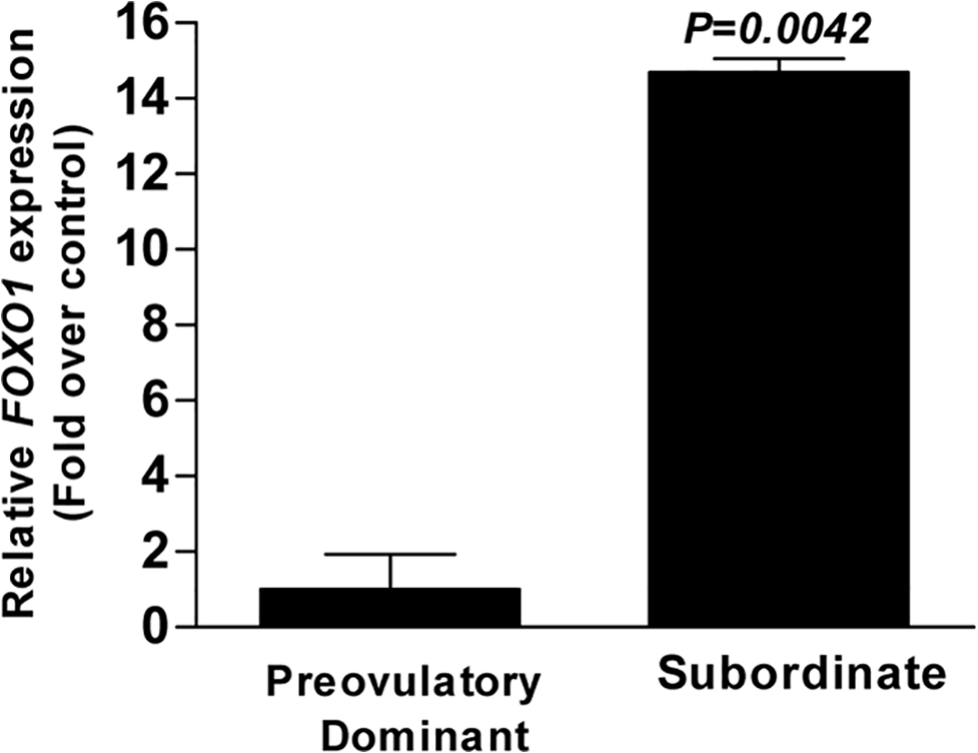
*FOXO1* is enriched in granulosa cells of subordinate follicles. The relative expression of *FOXO1*; a validated target of miR-183 cluster was quantified in granulosa cells of preovulatory dominant and subordinate follicles using qPCR. The relative expression of target transcript was normalized to the *GAPDH*. Statistical significance between groups was analyzed by two-tailed student t-test. Expression data is presented as mean ± SD of normalized Ct value of biological triplicates.

All members of the miR-183 cluster (bta-miR-183, bta-miR-96 and bta-miR-182) were the top three miRNAs highly enriched in granulosa cells of preovulatory dominant follicles. To find genes targeted by miR-183 cluster members, we used an online miRNA target gene prediction database; targetscan (http://www.targetscan.org/). All members the miR-183 cluster are predicted to co-ordinately target the 3´-UTR of bovine *FOXO1* gene (Fig 8). The 3,365 nucleotides long 3'-UTR of bovine *FOXO1* transcript contains binding sites for several miRNAs. To experimentally validate whether miR-183 cluster target the 3´-UTR of bovine *FOXO1* gene, we cloned a section of the 3´-UTR containing the putative miRNA binding sites into a dual luciferase reporter vector. We showed that, the luciferase firefly activity was significantly reduced upon co-transfection the *FOXO1* 3´-UTR plasmid construct with miR-183 cluster miRNA mimics (Fig 8). In contrast, co-transfection of miR-183 cluster miRNA mimics with *FOXO1* 3´-UTR mutant construct had no significant effect on the firefly luciferase activity (Fig 8). Interestingly, qPCR result showed that the expression level of *FOXO1* was highly enriched in granulosa cells of subordinate follicles compared to the preovulatory dominant follicles (Fig 9) and the expression profile of *FOXO1* gene showed reciprocal pattern to the expression of miR-183 cluster miRNAs.

## Discussion

The aim of this study was to investigate the expression pattern of regulatory miRNAs in granulosa cells derived from bovine preovulatory dominant and subordinate follicles using the Illumina deep sequencing approach and consequently to identify their potential role in bovine follicular development during the late follicular phase of bovine the estrous cycle. MiRNA deep sequencing quantifies the relative abundance of miRNAs by determining their frequencies in terms of read counts. Highly abundant miRNAs have higher likelihood of having higher read counts compared to miRNAs with lower abundance [34]. Of the total detected miRNAs, only 28 and 36 miRNAs were found to be specific to preovulatory and subordinate follicles, respectively while majority of the detected miRNAs (>80%) were commonly expressed between dominant and subordinate follicles. This implies that majority of the detected miRNAs may play a housekeeping role in maintaining normal physiological function in ovarian granulosa cells during the late follicular phase of the oestrus cycle. Interestingly, bta-miR-26a, bta-miR-10b and 3 isoforms of the let-7 family (bta-let-7a-5p, bta-let-7f and bta-let-7i) were among the top 10 abundantly expressed miRNAs in granulosa cells of both preovulatory dominant and subordinate follicles. We previously showed that these miRNAs are abundantly expressed in bovine granulosa cells derived from both dominant and subordinate follicle during early luteal phase of bovine estrous cycle [16]. Similarly, Miles et al. [35], applied cDNA libraries and massive parallel sequencing, showed members of the let-7 family to be abundantly expressed in bovine ovaries. Similarly, bta-miR-26a, let-7 family, bta-miR-10b and bta-miR-143 were among the top 10 abundantly expressed miRNAs in bovine ovarian and testicular tissues [36]. Previous study in our lab identified that Let-7 family and bta-miR-143 are abundantly expressed in cDNA library cloned from bovine ovary signifying their potential role in bovine ovarian functions [11].

In addition to the possibilities of quantifying the relative abundance of miRNAs, profiling the expression of miRNAs using next generation sequencing has greater advantage over other array based miRNome profiling tools (microarray and PCR panels) and direct sequencing of cDNA clones for its capability to predict novel unannotated miRNAs [23]. Currently there are 35,828 matured miRNAs discovered from 223 species deposited in the latest release of miRbase (release 21.0 June, 2014). The numbers of discovered bovine miRNAs are limited to 793, compared with 2,588 in human and 1,915 in mouse. We showed that 50–56% the sequence reads mapped to the reference bovine genome was mapped to known bovine miRNAs signifying that our sequencing libraries are predominantly enriched with miRNAs. Moreover, 11 novel miRNAs have been predicted from the sequence reads which were not mapped to reported matured miRNAs. In agreement with previous results [37], part of the remaining sequence reads that were not aligned to known miRNAs may represent novel miRNAs or other class of regulatory RNAs.

During miRNA biogenesis, a miRNA precursor is clipped by an endonuclease enzyme Drosha and exported into the cytoplasm for further processing by Dicer and give rise to a double stranded miRNA-duplex. The thermodynamic stability of each end of the miRNA-duplex determines the biologically active strand that can be incorporated into the miRNA-induced silencing complex (miRISC) while the other strand is considered to be inactive and subsequently be degraded [38]. Similarly, the asymmetric stability of the Dicer cleavage miRNA-duplexes suggests to determine the miRNA arm choice [39]. However, there are growing evidences indicating both arms of a miRNA precursor could be functional by targeting the same gene co-ordinately [40] and/or can have different targeting properties and different biological functions [41]. Our result demonstrated functional 3p and 5p arms of certain miRNA precursors. For instance, the 5p and 3p arms of mir-17 precursor were found to be expressed in both the preovulatory and subordinate follicles with slightly higher expression of the 5p arms. Both miR-17-5p and miR-17-3p have been implicated in co-ordinately targeting the *TIMP* metallopeptidase inhibitor 3 (*TIMP3*) gene and induce growth and invasion of prostate tumour [40]. We found the 3p arm of mir-22 to be highly abundant compared to its corresponding 5p arm with relatively higher abundance in preovulatory dominant follicles and it also reported to inhibits the Estrogen signaling pathways by inhibiting the expression of Estrogen Receptor (*ERα*) mRNA [42]. This is further supported by the fact that there a sharp decline in estradiol concentration in the circulation during the preovulatory period [43]. Thus, it can be assumed that the 3p arm of bta-mir-22 precursor is functionally more relevant than the corresponding 5p arm during the late follicular phase of preovulatory stage of bovine estrous cycle.

Differential expression of certain miRNAs in different stages of bovine follicular developments and cell types may provide valuable insight into their potential role in folliculogenesis in stage manner. Both bta-miR-21-3p and bta-miR-21-5p are enriched in granulosa cells of preovulatory dominant follicle with 3.6 and 2.1-folds higher, respectively. Moreover, bta-miR-21-5p was among the top 10 miRNAs abundantly expressed in preovulatory dominant follicles with an average read counts of 8,695. Up-regulation of miR-21 in mouse luteinizing mural granulosa cells was reported following human chorionic gonadotropin (hCG) treatment [44]. Increased cell apoptosis was observed in mural granulosa cells transfected with miR-21 targeting LNA oligonucleotide (miR21-LNA) evidencing the critical role of miR-21 in preventing apoptosis of granulosa cells of preovulatory follicle following the LH surge. Moreover, in-vivo experiments demonstrated that there was significant reduction in ovulation rate and subsequently low number of cumulus-oocyte-complex recovery in oviducts of miR21-LNA inhibitor treated ovaries compared to the untreated controls. This implies that bta-miR-21 plays important role in regulating bovine follicular development and preventing apoptosis of granulosa cells by targeting the 3´-UTR of proappototic genes in preovulatory dominant follicles.

Similarly, the miR-132 family (bta-miR-132 and bta-miR-212) was among the robustly expressed miRNAs in granulosa cells of preovulatory dominant follicles with 60 and 26-fold higher, respectively compared to the subordinate follicles counterparts. Bta-miR-132 and bta-miR-212 are transcribed from intergenic region of chromosome 19 of the bovine genome and have the same seed region. Fiedler et al. [14] demonstrated that up regulation of both miR-132 and miR-212 in mouse preovulatory granulosa cells following the induction of ovulatory dose of LH/hCG. Inhibiting the expression of both miR-132 and miR-212 using LNA inhibitor showed increased the C-terminal binding protein-1 (*CTBP1*) protein levels. Interestingly, *CTBP1* regulates adrenal steroidogenesis by periodically interacting with steroidogenic factor 1 (*SF1*), which in turn regulates the transcription of *CYP17* gene [14].

We showed bta-miR-378 to be 4-folds lower in granulosa cells of preovulatory dominant follicles in comparison with the subordinate ones. MiR-378 targets the 3´-UTR of aromatase gene *(CYP19A)*; a gene responsible for estradiol biosynthesis in granulosa cells and inhibition of miR-378 in vitro resulted an increased estradiol production implying aromatase gene is post-transcriptionally regulated by the action of miR-378 [45]. Thus, down regulation of miR-378 in preovulatory dominant follicles may suggest increased level of aromatase gene. Similarly, bta-let-7f as one of the eight let-7 family isoforms, found to be highly abundant both in dominant and subordinate follicles with slightly higher expression in subordinate follicles. The let-7f is reported as a tumour suppressor miRNA in breast cancer cells and further validated to target *CYP19A* gene [46].

Members of the miR-183 cluster; bta-miR-96, bta-miR-182 and bta-miR-183 are transcribed from intergenic region of chromosome 4 of the bovine genome. All members of the miR-183 cluster were found to be the top 3 highly enriched miRNAs in granulosa cells of preovulatory dominant follicles with fold regulation of 130.7, 89.9 and 85.7, respectively. We showed that these list of conserved miRNAs target the 3´-UTR *FOXO1*gene; a transcription factor which induces expression of genes involved in apoptosis, glucose metabolism, cell cycle progression and differentiation [47]. Similarly the expression of the apoptotic *FOXO1* gene showed marked reduction in granulosa cells preovulatory dominant follicles showing opposite expression pattern with miR-183 cluster miRNAs. This could signify that enrichment of *FOXO1* in subordinate follicles could facilitate the activation of proapoptotic downstream target genes which in turn play role in follicular atresia. In consistence with our finding, Shi and LaPolt [48] showed decreased expression of *FOXO1* protein in granulosa cells of healthy preovulatory follicles compared to the follicles under going atresia. Similarly, increased *FOXO1* expression in mouse granulosa cells has been implicated with accumulation of reactive oxygen species (ROS) [49]. Following the LH surge, numerous genes related to inflammation are expressed in preovulatory follicles which leads to massive recruitment of ROS, macrophages and neutrophils from inflammatory cells [50]. Depletion of both neutrophils and macrophages can reduce ovulation in rat and mouse ovaries [51,52]. Thus, further *in-vitro* functional study is needed to confirm whether the miR-183 cluster in granulosa cells of preovulatory dominant follicles co-ordinately suppress the expression of *FOXO1* and other downstream proapoptotic genes and prevent apoptosis of granulosa cells. Furthermore, the role of miR-183 cluster in ROS accumulation in granulosa cells is yet to be determined.

Expression of selected miRNAs in theca cell, COC showed that the relative abundance of the member of the miR-183 and miR-132 cluster is higher in preovulatory dominant follicles. This supports the crosstalk between granulosa cells and other companion cells within the follicle. Furthermore, increased miRNAs level in follicular fluids could further be supported by the fact that there are significant number of circulatory miRNAs in bovine follicular fluid carried by exosomes and have potential role in cell-to-cell communication in follicular microenvironment [53].

Pathways known to be involved in ovarian functions and hormonal regulation namely; Axon guidance, MAPK signalling pathway, Wnt signaling pathways, TGF-β signaling pathway, GnRH signaling pathways and progesterone-mediated oocyte maturation were among the highly enriched canonical pathways enriched by predicted target genes of differentially expressed miRNAs. For instance, the Wnt signaling pathway is enriched by 10 up-regulated and 10 down-regulated miRNAs and is known to be involved in mammalian reproduction including follicular development, ovulation, formation and regression of Paramesonephric duct [54]. Similarly, the *Wnt-4* gene regulates the function of ovarian granulosa cells in rodent ovary in stage specific expression of specific *Wnt/Fz* genes [55]. Interestingly, ErbB signalling pathway was enriched by only target genes of miRNAs up-regulated in preovulatory dominant follicles (bta-miR-129-5p, bta-miR-221, bta-miR-339b and bta-miR-96) and suggests possible involvement of these miRNAs in modulating ErbB gene family during bovine follicular development. Similarly, apoptosis pathway was enriched by only target genes of miRNAs down-regulated in preovulatory dominant follicles (bta-miR-1271, bta-miR-17-5p and bta-miR-365-3p). This signifies enrichment of specific miRNAs in subordinate follicles could trigger apoptosis pathways by post-transcriptionally regulating the balance between pro and anti-apoptotic genes to determine the fate of follicular cells and cause follicular atresia.

## Conclusion

The spatio-temporal expression of miRNAs in granulosa cells during the follicular phase of the estrous cycle supports the potential role of miRNAs in post-transcriptional regulation of genes involved in bovine follicular development, mainly ovulation of a preovulatory dominant follicle and regression of anovulatory subordinate follicles. In addition to differential expression of miRNAs, the present study identified cluster of miRNAs which are abundantly expressed in granulosa cells of both preovulatory and regressing follicles signifying their housekeeping role during follicular development. The information we provided here may be helpful in deciphering the molecular mechanism of bovine follicular ovulation and atresia. Further *In-vitro* experiment is required to fully understand the specific functional role of classes or cluster of miRNAs during various stages of the follicular development in general and the follicular phase of the estrous cycle in particular.

## Supporting Information

S1 Fig Purity of granulosa cells check-up using granulosa cells-specific gene markers. Granulosa cell-specific marker gene (*FSHR*) was detected in both dominant and subordinate follicles at higher level as indicated by strong bands, while theca cell-specific marker gene (*CYP17A1*) had weaker band. Efficiency of cDNA synthesis was confirmed using housekeeping *GAPDH* gene. Legend: S1, S2, S3 and D1, D2, D3 represent granulosa cell samples derived from subordinate and preovulatory dominant follicles of day 19 of the estrous cycle, respectively.
(TIF)

S2 Fig Read count distribution of detected miRNAs in libraries of preovulatory dominant and subordinate follicles. (TIF)

S3 Fig Graphic illustration of a representative miRNA precursor (bta-mir-126) with functional 5p and 3p arms. (TIF)

S1 Table List of adaptors and primers used during library construction, PCR amplification of target genes and luciferase reporter assay. (DOCX)

S2 Table List of all detected miRNAs in granulosa cells of preovulatory dominant and subordinate follicles. Normalized read count of each miRNAs in biological triplicates are indicated.
(XLSX)

S3 Table Top 20 gene ontology terms enriched by predicted target genes of differentially expressed miRNAs in preovulatory dominant follicles. (DOCX)

S4 Table KEGG pathway analysis, list of representative pathways known to be involved in follicular development and related physiologies enriched by predicted target genes. List of all miRNAs predicted to be involved in each pathway are indicated. MiRNAs highlighted in bold and italic are up and down regulated in preovulatory dominant follicles, respectively.
(DOCX)
