# Supplementary figures and images for: MicroRNA Expression Profile in Bovine Granulosa Cells of Preovulatory Dominant and Subordinate Follicles during the Late Follicular Phase of the Estrous Cycle
Source: PLoS One. 2015 May 19;10(5):e0125912. doi: 10.1371/journal.pone.0125912 (PMC4438052; doi:10.1371/journal.pone.0125912)

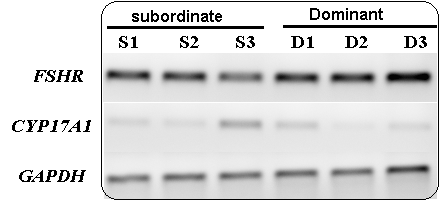

Supplement: S1 Fig — Granulosa cell-specific marker gene (FSHR) was detected in both dominant and subordinate follicles at higher level as indicated by strong bands, while theca cell-specific marker gene (CYP17A1) had weaker band. Efficiency of cDNA synthesis was confirmed using housekeeping GAPDH gene. Legend: S1, S2, S3 and D1, D2, D3 represent granulosa cell samples derived from subordinate and preovulatory dominant follicles of day 19 of the estrous cycle, respectively. (TIF) [file pone.0125912.s001.tif]

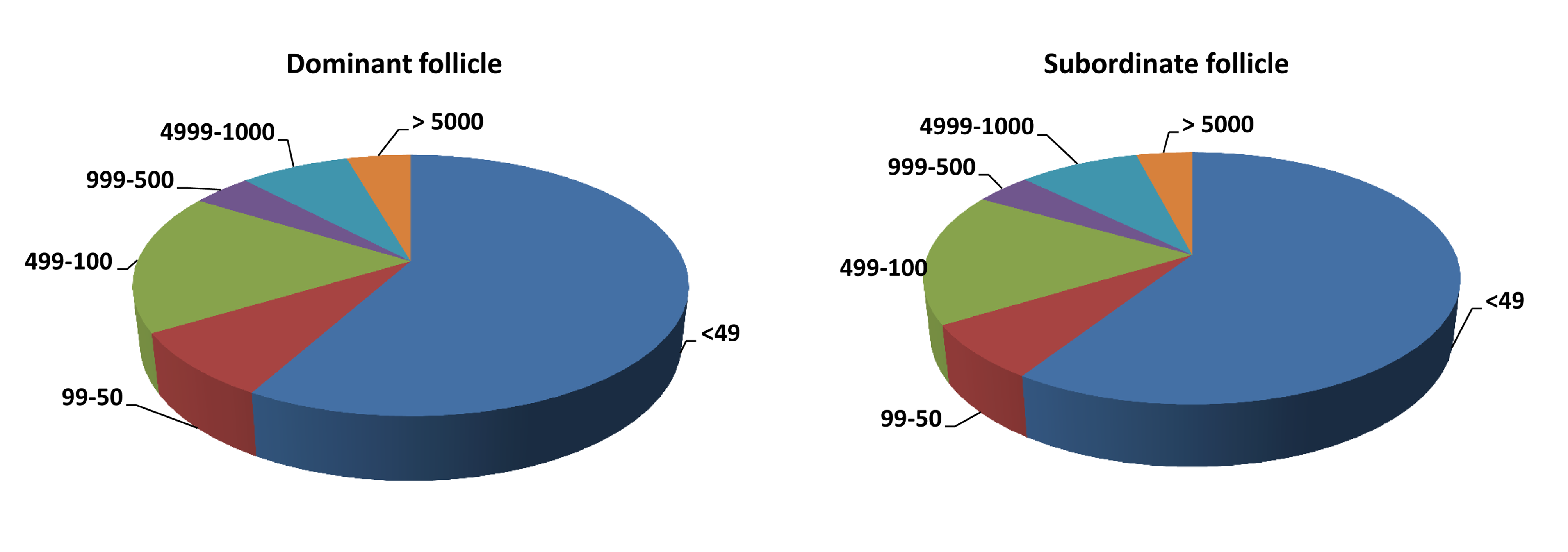

Supplement: S2 Fig — (TIF) [file pone.0125912.s002.tif]

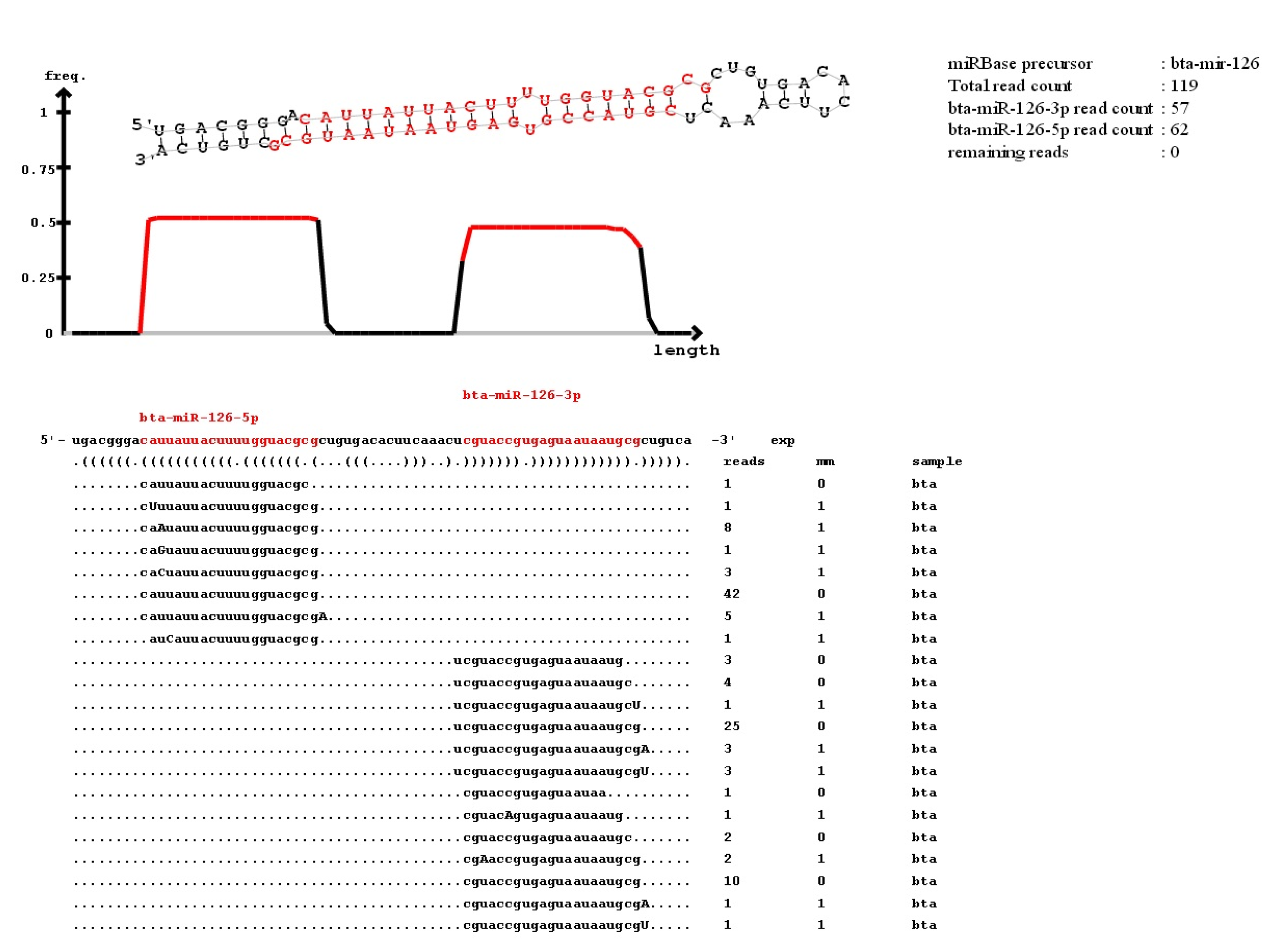

Supplement: S3 Fig — (TIF) [file pone.0125912.s003.tif]
