# Supplementary material for: MicroRNA Expression Profile in Bovine Granulosa Cells of Preovulatory Dominant and Subordinate Follicles during the Late Follicular Phase of the Estrous Cycle
Source: PLoS One. 2015 May 19;10(5):e0125912. doi: 10.1371/journal.pone.0125912 (PMC4438052; doi:10.1371/journal.pone.0125912)
Supplement: S1 Table — (DOCX) [file pone.0125912.s004.docx]

**Table S1: List of primers and adaptors used during library construction, PCR amplification and luciferase assay**

List f primers for semi Quantitative and Quantitative PCR

| **Gene Name** | | **Sequence (5´-3´)** | **Tm (^O^C)** | **Accession no.** |
| --- | --- | --- | --- | --- |
| *GAPDH* | For.  Rev. | AATGGAGCCATCACCATC  GTGGTTCACGCCCATCACA | 55 | NM_001034034 |
| *FSHR* | For.  Rev. | GCAGTCGAACTGAGGTTTGT  AGATATCGGAGGTTGGGAAG | 55 | NM_174061 |
| *CYP17A1* | For.  Rev. | CTACTTGCCCTTTGGAGCAG  GGAGTCATGAGGTGCTACCC | 57 | NM_174304 |
| *FOXO1* | For.  Rev. | AAGAGCGTGCCCTACTTCAA  CTCTTCTCCTGGGGGATTTC | 57 | XM_583090 |

List of Adapters and primers used for library construction

| **Adapter/Primer** | **Sequence (5´-3´)** |
| --- | --- |
| RNA 5´ adapter | GTTCAGAGTTCTACAGTCCGACGATC |
| RNA 5´ adapter (RC) | GATCGTCGGACTGTAGAACTCGAAC |
| RNA 3´ adapter | TGGAATTCTCGGGTGCCAAGG |
| RNA 3´ adapter (RC) | CCTTGGCACCCGAGAATTCCA |
| RNA PCR primer | AATGATACGGCGACCACCGAGATCTACACGTTCAGAGTTCTCCAGTCCGA |
| RNA PCR primer (RC) | TCGGACTGTAGAACTCTGAACGTGTAGATCTCGGTGGTCGCCGTATCATT |
| RNA Reverse transcription primer | GCCTTGGCACCCGAGAATTCCA |
| RNA Reverse transcription primer (RC) | TGGAATTCTCGGGTGCCAAGGC |

RC: reverse complement For: forward primer Rev: reverse primer

**List of primers used for luciferase assay**

| **Oligo name** | **Sequence (5´-3´)** | **Tm (^O^C)** |
| --- | --- | --- |
| Wild-type FOXO1-3´-UTR-Forward | CGTGGAGCTCTCCTTTCGTCAGACTTGGCA | 70 |
| Wild-type FOXO1-3´-UTR-Reverse | GACTCTCGAGGCACACCAGGATCCAAAAGTC | 70 |
| Mutant FOXO1-3´-UTR-Forward | CGTGGAGCTCCAGATAAGGACTTCAGTCGTGGAAATTCATCTCGAGGACT | 75 |
| Mutant FOXO1-3´-UTR-Reverse | AGTCCTCGAGATGAATTTCCACGACTGAAGTCCTTATCTGGAGCTCCACG | 75 |
