# Supplementary material for: MicroRNA Expression Profile in Bovine Granulosa Cells of Preovulatory Dominant and Subordinate Follicles during the Late Follicular Phase of the Estrous Cycle
Source: PLoS One. 2015 May 19;10(5):e0125912. doi: 10.1371/journal.pone.0125912 (PMC4438052; doi:10.1371/journal.pone.0125912)
Supplement: S3 Table — (DOCX) [file pone.0125912.s006.docx]

Table S3. Top 20 GO terms enriched by predicted target genes differentially expressed preovulatory dominant follicles.

| **Target genes of Up Regulated miRNAs** | | **Target genes of Down Regulated miRNAs** | |
| --- | --- | --- | --- |
| **GO Term** | **Enrichment P Value** | **GO Term** | **Enrichment P Value** |
| GO:0042127~ regulation of cell proliferation | 1.37E-12 | GO:0042127~ regulation of cell proliferation | 3.48E-19 |
| GO:0010604~ positive regulation of macromolecule metabolic process | 3.06E-10 | GO:0010604~ positive regulation of macromolecule metabolic process | 3.86E-15 |
| GO:0043067~ regulation of programmed cell death | 1.06E-09 | GO:0010557~ positive regulation of macromolecule biosynthetic process | 2.54E-12 |
| GO:0010941~ regulation of cell death | 1.16E-09 | GO:0051254~ positive regulation of RNA metabolic process | 4.44E-12 |
| GO:0042981~ regulation of apoptosis | 3.11E-09 | GO:0031328~ positive regulation of cellular biosynthetic process | 6.56E-12 |
| GO:0043069~ negative regulation of programmed cell death | 3.95E-09 | GO:0009891~ positive regulation of biosynthetic process | 8.81E-12 |
| GO:0060548~ negative regulation of cell death | 4.16E-09 | GO:0045893~ positive regulation of transcription, DNA-dependent | 3.51E-11 |
| GO:0008284~ positive regulation of cell proliferation | 1.10E-08 | GO:0045935~ positive regulation of nucleobase, nucleoside, nucleotide and nucleic acid metabolic process | 6.35E-11 |
| GO:0043066~ negative regulation of apoptosis | 1.62E-08 | GO:0051173~ positive regulation of nitrogen compound metabolic process | 1.14E-10 |
| GO:0048534~ hemopoietic or lymphoid organ development | 1.74E-08 | GO:0045944~ positive regulation of transcription from RNA polymerase II promoter | 4.92E-10 |
| GO:0030097~ hemopoiesis | 2.46E-08 | GO:0045941~ positive regulation of transcription | 6.06E-10 |
| GO:0002520~ immune system development | 4.36E-08 | GO:0030182~ neuron differentiation | 6.62E-10 |
| GO:0001501~ skeletal system development | 7.56E-08 | GO:0007423~ sensory organ development | 9.05E-10 |
| GO:0042325~ regulation of phosphorylation | 9.72E-08 | GO:0010628~ positive regulation of gene expression | 9.96E-10 |
| GO:0045597~ positive regulation of cell differentiation | 9.88E-08 | GO:0006357~ regulation of transcription from RNA polymerase II promoter | 1.05E-09 |
| GO:0019220~ regulation of phosphate metabolic process | 1.99E-07 | GO:0042325~ regulation of phosphorylation | 1.70E-09 |
| GO:0051174~ regulation of phosphorus metabolic process | 1.99E-07 | GO:0043066~ negative regulation of apoptosis | 2.34E-09 |
| GO:0045596~ negative regulation of cell differentiation | 2.73E-07 | GO:0008284~ positive regulation of cell proliferation | 2.41E-09 |
| GO:0007423~ sensory organ development | 5.78E-07 | GO:0043069~ negative regulation of programmed cell death | 2.83E-09 |
| GO:0031328~ positive regulation of cellular biosynthetic process | 6.53E-07 | GO:0060548~ negative regulation of cell death | 2.94E-09 |
