# Supplementary material for: MicroRNA Expression Profile in Bovine Granulosa Cells of Preovulatory Dominant and Subordinate Follicles during the Late Follicular Phase of the Estrous Cycle
Source: PLoS One. 2015 May 19;10(5):e0125912. doi: 10.1371/journal.pone.0125912 (PMC4438052; doi:10.1371/journal.pone.0125912)
Supplement: S4 Table — List of all miRNAs predicted to be involved in each pathway are indicated. MiRNAs highlighted in bold and italic are up and down regulated in preovulatory dominant follicles, respectively. (DOCX) [file pone.0125912.s007.docx]

Table S4. Representative list of pathways related to follicular development enriched by target genes of differentially expressed miRNAs

| Pathway | Predicted target genes involved in pathway ¥ | List of DE miRNAs involved in pathway | *p*-value* |
| --- | --- | --- | --- |
| ***Axon guidance*** | DPYSL2, CFL2, SEMA5A, SEMA4A, SEMA3, SEMA4A, SEMA6A, RAC1, RAC2, PPP3CC, PPP3R1, PAK1, PAK2, PAK6, MET, PLXNA2 | **bta-miR-132, bta-miR-152, bta-miR-182, bta-miR-183, bta-miR-204, bta-miR-212, bta-miR-221, bta-miR-29b, bta-miR-339b, bta-miR-375, bta-miR-424-5p, bta-miR-96,** *bta-miR-1271, bta-miR-17-5p, bta-miR-19a, bta-miR-20a, bta-miR-214, bta-miR-296, bta-miR-409a, bta-miR-449a, bta-miR-769* | 0.037 to 3,58E+12 |
| ***Wnt signaling pathway*** | FZD4, FZD1, FZD6, WNT9A, WNT3A, FZD7, WNT7A, LRP6, FZD2, WNT8B, SMAD4, WNT1, WNT11, TBL1XR1, TCF7L1 | **bta-miR-129-5p, bta-miR-132, bta-miR-152, bta-miR-182, bta-miR-183, bta-miR-204, bta-miR-212, bta-miR-421, bta-miR-424-5p, bta-miR-96,** *bta-miR-10b, bta-miR-1271, bta-miR-17-5p, bta-miR-20a, bta-miR-214, bta-miR-296, bta-miR-409a, bta-miR-449a* | 0.041 to 7,07E+09 |
| ***MAPK signaling pathway*** | MAP4K3, MAP3K14, MAP3K3, MAP4K4, IKBKB, TRAF2, TGFBR1, TNFRSF1A, BDNF, FGF1, FGF2, CACNB3, CACNB4, PDGFRA, GRB2, NRAS, MRAS, KRAS, RRAS. RASA1 | **bta-miR-182, bta-miR-204, bta-miR-22-3p, bta-miR-424-5p, bta-miR-873, -bta-miR-96*,*** *bta-miR-1271, bta-miR-1296, bta-miR-17-5p, bta-miR-20a, bta-miR-2240, bta-miR-296, bta-miR-365-3p* | 0.046 to 1,15E+12 |
| ***Progesterone-mediated oocyte maturation*** | IGF1, GNAI1, GNAI2, PIK3R1, PRKACA, PRKACB, PIK3R3, RPS6KA3, CDC23, CPEB1, MAPK3 | **bta-miR-182, bta-miR-424-5p, bta-miR-96**, *bta-miR-19a, bta-miR-296, bta-miR-365-3p, bta-miR-99a, bta-miR-99b* | 0,028 to 1,40E+12 |
| ***Calcium signaling pathway*** | ADCY1, ADCY7, PRKACB, CACNA1H, CACNA1I, CACNA1A, ITPR1, ATP2A3, GNAQ, RYR2 | **bta-miR-129-5p, bta-miR-182, bta-miR-204, bta-miR-96,** *bta-miR-19a, bta-miR-296* | 0.045 to 0.002 |
| ***Oocyte meiosis*** | IGF1, ADCY1, ADCY7, IGF1R, PRKACA, SMC1A, PPP2R5C, ADCY6, ADCY2, CPEB1, MAP2K1, PPP2R5E | **bta-miR-132, bta-miR-182, bta-miR-424-5p, bta-miR-96,** *bta-miR-19a* | 0.033 to 0.001 |
| ***GnRH signaling pathway*** | FSHB, GNAS, ITPR2, PLCB2, GNAQ | **bta-miR-182, bta-miR-96*,*** *bta-miR-1271, bta-miR-19a, bta-miR-296* | 0,024 to 4,88E+12 |
| ***TGF-beta signaling pathway*** | BMP8B, TGFBR1, BMPR2, GDF6, SMAD6, SMAD2, RBL1, TGFBR2, SMAD7, NOG, E2F5, BMP2,  SKP1 | **bta-miR-152, bta-miR-183**, *bta-miR-17-5p, bta-miR-20a, bta-miR-214* | 0,042 to 1,59E+12 |
| ***ErbB signaling pathway*** | PIK3R1, STAT5B, SHC1, ERBB4, SHC4, GRB2 | **bta-miR-129-5p, bta-miR-221, bta-miR-339b, bta-miR-96** | 0.045 to 4,53E+11 |
| ***Cell cycle*** | GADD45A, CCND1, RBL1, RBL2, RB1, WEE1, CCND3, E2F3, CCND2 | **bta-miR-152, bta-miR-424-5p, bta-miR-450b,** *bta-miR-20a* | 0.049 to 0.010 |
| ***Apoptosis*** | AKT3, CASP9, BCL2, XIAP, TNFRSF1A, CASP8, CASP7, IRAK4 | *bta-miR-1271, bta-miR-17-5p, bta-miR-365-3p* | 0.041 to 0,014 |
| ***p53 signaling pathway*** | CDK6, CDKN1A, CHEK1, BAI1, CCND3, PTEN, SHISA5 | **bta-miR-202, bta-miR-424-5p**, *bta-miR-20a* | 0.042 to 0.021 |
| ***RNA Degradation*** | PAPOLB, PAPOLA, CNOT6L, PAPOLG,  CNOT7, XRN1, CNOT6, CNOT4,  PATL1, DCP2, DDX6 | *bta-miR-17-5p, bta-miR-19a* | 0.027-0.025 |
| ***Hedgehog signaling***  ***pathway*** | WNT4, GSK3B, PTCH1 | *bta-miR-769* | 0.018 |
| ***VEGF signaling pathway*** | MAPK1, KRAS,  NFAT5, PPP3CC, PXN | **bta-miR-132** | 0.078 |
| ***Jak-STAT signaling***  ***pathway*** | IL2RB, SOCS2, STAM2, CBL, LIFR,  SOCS4, SOCS5, STAT1, IL10, PTPN11  LIF, SPRY1, NTF, CLCF1, SPRED2  IL2RG, IL12B, PIK3R3, AKT3, GHR | **bta-miR-421** | 0.0061 |

¥: Only representative predicted genes are presented

*: Ranges of p-values for genes involved in specific pathway
